# Identification and Discrimination of *Salmonella enterica* Serovar Gallinarum Biovars Pullorum and Gallinarum Based on a One-Step Multiplex PCR Assay

**DOI:** 10.3389/fmicb.2018.01718

**Published:** 2018-07-31

**Authors:** Dan Xiong, Li Song, Zhiming Pan, Xinan Jiao

**Affiliations:** ^1^Jiangsu Key Laboratory of Zoonosis, Yangzhou University, Yangzhou, China; ^2^Jiangsu Co-innovation Center for Prevention and Control of Important Animal Infectious Diseases and Zoonoses, Yangzhou University, Yangzhou, China; ^3^Key Laboratory of Prevention and Control of Biological Hazard Factors (Animal Origin) for Agri-Food Safety and Quality, Ministry of Agriculture of China, Yangzhou University, Yangzhou, China; ^4^Joint International Research Laboratory of Agriculture and Agri-Product Safety of the Ministry of Education, Yangzhou University, Yangzhou, China

**Keywords:** *Salmonella* Pullorum, *Salmonella* Gallinarum, multiplex PCR, accurate discrimination, one-step diagnostic PCR

## Abstract

*Salmonella enterica* serovar Gallinarum biovars Pullorum (*S.* Pullorum) and Gallinarum (*S.* Gallinarum) can result in pullorum disease and fowl typhoid in avian species, respectively, and cause considerable economic losses in poultry in many developing countries. Conventional *Salmonella* serotyping is a time-consuming, labor-intensive and expensive process, and the two biovars cannot be distinguished using the traditional serological method. In this study, we developed a rapid and reliable one-step multiplex polymerase chain reaction (PCR) assay to simultaneously identify and discriminate the biovars Pullorum and Gallinarum. The multiplex PCR method focused on three specific genes, *stn*, *I137_08605* and *ratA*. Based on bioinformatics analysis, we found that gene *I137_08605* was present only in *S.* Pullorum and *S.* Gallinarum, and a region of difference in *ratA* was deleted only in *S.* Pullorum after comparison with that of *S.* Gallinarum and other *Salmonella* serovars. Three pairs of primers specific for the three genes were designed for the multiplex PCR system and their selectivity and sensitivity were determined. The multiplex PCR results showed that *S.* Pullorum and *S.* Gallinarum could be identified and discriminated accurately from all tested strains including 124 strains of various *Salmonella* serovars and 42 strains of different non-*Salmonella* pathogens. In addition, this multiplex PCR assay could detect a minimum genomic DNA concentration of 67.4 pg/μL, and 100 colony forming units. The efficiency of the multiplex PCR was evaluated by detecting natural-occurring *Salmonella* isolates from a chicken farm. The results demonstrated that the established multiplex PCR was able to identify *S.* Gallinarum and *S.* Pullorum individually, with results being consistent with traditional serotyping and biochemical testing. These results demonstrated that a highly accurate and simple biovar-specific multiplex PCR assay could be performed for the rapid identification and discrimination of *Salmonella* biovars Gallinarum and Pullorum, which will be useful, particularly under massive screening situations.

## Introduction

Salmonellosis is a zoonotic disease, which can cause gastroenteritis, diarrhea, and systemic typhoid fever (McGhie et al., 2009). So far, 2,610 different *Salmonella* serovars have been defined and classified based on the 46 lipopolysaccharide (O) and 114 flagellar (H) antigens (Guibourdenche et al., 2010). *Salmonella enterica* serovar Gallinarum biovars Pullorum (*S.* Pullorum) and Gallinarum (*S.* Gallinarum) belong to biovars of serovar Gallinarum within serotype D, and cause diseases only in avian species including turkeys, chickens, and other birds (Shivaprasad, 2000).

*Salmonella* Gallinarum is the causative pathogen of fowl typhoid and results in variable morbidity and high mortality, which often leads to a severe septicemic disease occurring primarily in adult birds. *S.* Pullorum can cause pullorum disease, which is a serious systemic disease with high mortality, especially in young birds (Barrow and Freitas Neto, 2011). Although part of infected birds could get recovered from pullorum disease, and some adult birds may not present with any clinical disease symptoms, most birds develop a carrier state, and thus may become a repository for the pathogens, which may then be transmitted vertically to newborn hatchlings and horizontally to other birds (Berchieri et al., 2001; Shivaprasad and Barrow, 2008). Although these diseases have been wiped out from the commercial poultry industries in some developed countries, they are widespread in developing countries such as Asia, Africa, and Central and South America, and have caused enormous economic losses (Jones et al., 2001; Kang et al., 2011).

To date, the only reliable method for *Salmonella* serotyping is the traditional White–Kauffmann–Le Minor scheme, but it cannot distinguish closely related biovars, such as *S.* Gallinarum and *S.* Pullorum (World Organization for Animal Health (OIE), 2012). These two biovars share many similar characteristics, including the same lipopolysaccharide (O) antigens, the lack of flagella because of mutations occurred in flagellar-associated genes, and the same avian host they infect (Barrow and Freitas Neto, 2011). Thus, it is not possible to differentiate the two biovars only by serotyping. Although very similar antigenic features are shared by the two *Salmonella* biovars, some biochemical reactions such as glucose, maltose, and dulcitol fermentation, and ornithine decarboxylase activity, have been established and used to differentiate them (Crichton and Old, 1990). However, biochemical reactions are labor-intensive and take approximately 2–3 days to obtain results (Cheraghchi et al., 2014).

In recent years, polymerase chain reaction (PCR) has been rapidly developed and widely used for the detection of different pathogens because of its relatively simple procedures, as well as its potent specificity and sensitivity (Hoorfar, 2011). DNA-based molecular technologies have also been reported for use in the identification of *Salmonella* biovars Pullorum and Gallinarum (Kisiela et al., 2005; Kang et al., 2011). However, these assays to identify the two biovars are laborious, complicated, and expensive due to the requirement of additional reagents such as restriction enzymes, or the need of more than one single reaction.

In this study, a reliable and rapid multiplex PCR method were developed and validated to identify and distinguish *S.* Gallinarum and *S.* Pullorum simultaneously. The approach was based on three primer pairs designed to target specific regions of *stn*, *I137_08605*, and *ratA*, respectively. The multiplex PCR selectivity was evaluated among various *Salmonella* serovars and different non-*Salmonella* pathogens. Besides, natural-occurring *Salmonella* isolates from a commercial chicken farm were tested by the PCR assay. The newly established multiplex PCR method will be a useful tool to accurately identify and distinguish the two specific *Salmonella* biovars, and will be particularly beneficial in situations requiring massive screening.

## Materials and Methods

### Bacterial Strains

Bacterial strains used in this study are commercially available or were isolated previously after our routine monitoring, and included 124 strains which belong to 31 various *Salmonella* serovars and 42 strains of different non-*Salmonella* pathogens. All *Salmonella* serotypes were confirmed according to the White–Kauffmann–Le Minor scheme (Grimont and Weill, 2007), based on the rapid agglutination assays with the *Salmonella* diagnostic antisera (Tianrun Bio-Pharmaceutical, Ningbo, China). Ornithine decarboxylation and dulcitol fermentation tests were conducted to differentiate the biovars Gallinarum and Pullorum.

### Bacterial Culture and Genomic DNA Preparation

The bacterial growth and genomic DNA preparation were conducted as described previously (Xiong et al., 2016). In brief, frozen stocks of the bacterial strains were recovered on Luria-Bertani (LB) agar (Oxoid, Basingstoke, Hampshire, United Kingdom) or Brain Heart Infusion (BHI) agar (Becton, Dickinson and Company, Sparks, MD, United States) at 37°C overnight. The colonies were transferred to LB broth or BHI broth, and cultured at 37°C with constant shaking at a speed of 180 rpm for 16 h. Bacterial genomic DNA was prepared by a commercial QIAamp DNA Mini Kit (Qiagen, Hilden, Germany). The DNA purity and concentration were evaluated by the spectrophotometer NanoDrop ND-1000 (Thermo Scientific, Wilmington, DE, United States).

### Bioinformatics Analysis

To detect and differentiate *S.* Gallinarum and *S.* Pullorum based on a PCR assay, we exploited the basic local alignment search tool algorithm from the National Center for Biotechnology Information. The *I137_08605* (GenBank accession no. CP006575.1 segment 1847407-1847696) and *ratA ROD* (GenBank accession no. AM933173.1 segment 2636413-2636983) genes were each used in searches of the non-redundant nucleotide collection (nr/nt) database. To ensure that all aligned target sequences in the database were displayed, we set the number of nucleotide sequences to the maximum value of 20,000. Two pairs of primers specific for the two genes were designed using the online software Primer3 (Untergasser et al., 2012), and the specific primer pairs for the targets are listed in **Table 1**.

**Table 1:** Multiplex PCR primers used for identification and discrimination of *S.* Pullorum and *S.* Gallinarum.

Primers	Primer sequence (5′→3′)	Size (bp)	Accession no./Nt segments	*Salmonella* serovars	
				SP	SG
*stn* F	TATTTTGCACCACAGCCAGC	131	L16014.1 450–580	+	+
*stn* R	CGACCGCGTTATCATCACTG				
*I137_08605* F	CACTGGAGACTCTGAGGACA	290	CP006575.1 1847407-1847696	+	+
*I137_08605* R	GGGCAGGGAGTCTTGAGATT				
*ratA ROD* F	ATTGCTCTCGTCCTGGGTAC	571	AM933173.1 2636413-2636983	-	+
*ratA ROD* R	TACCGATACGCCCAACTACC				

*SP, *S.* Pullorum; SG, *S.* Gallinarum*.

### Multiplex PCR Procedure

The multiplex PCR assays were performed in a final volume of 25-μL including 200 μM deoxynucleoside triphosphate mix, 0.25 μL (1 U) of Taq DNA polymerase, 2.5 μL of 10× Taq DNA polymerase buffer (Vazyme, Nanjing, China), 40 nM of the *ratA ROD* F/R primers, 80 nM of the *I137_08605* F/R primers, 80 nM of the *stn* F/R primers, and 100 ng of the bacterial genomic DNA. The *stn* gene encodes *Salmonella* enterotoxin and is specific for *S. enterica* (Prager et al., 1995). Therefore, *stn* could be used as the internal amplification control in the multiplex PCR method. The PCR amplification was conducted using a T100 Thermal Cycler (Eppendorf, Hamburg, Germany) with the following protocol: initial denaturation at 94°C for 5 min; 30 sequential cycles of 94°C for 45 s, 52°C for 45 s, and 72°C for 40 s; and a final step of 72°C for 10 min. The PCR fragments were separated using 1% agarose gels after electrophoresis, and stained with 1× GelRed Nucleic Acid Gel Stain (Biotium, Fremont, CA, United States). The amplified PCR products were visualized using UV light excitation, and images were digitalized using a GelDoc XR Gel Documentation System (Bio-Rad).

### Selectivity and Detecting Limit of the Multiplex PCR Assay

Genomic DNA extracted from 124 strains of various *Salmonella* serovars and 42 strains of different non-*Salmonella* pathogens were used to evaluate the selectivity and compatibility of the three primer sets in the multiplex PCR assay.

The detecting limit of the multiplex PCR assay regarding the lower limit of DNA that could be detected was determined by testing serially diluted samples of genomic DNA from *S.* Gallinarum strain SG9 and *S.* Pullorum strain S06004. The genomic DNA of the two *Salmonella* biovars was 10-fold serially diluted from 67.4 ng/μL to 674 fg/μL in deionized water. All diluted samples were separately served as the template in the established multiplex PCR assay.

To determine the fewest number of cells that could be detected by the multiplex PCR, *S.* Gallinarum strain SG9 and *S.* Pullorum strain S06004 were cultured overnight. The bacteria were centrifuged and washed with phosphate buffered saline three times, and the plate counting assays were used to calculate the bacterial concentrations. The templates were prepared by 10-fold serially dilution to obtain different concentrations of bacteria ranging from 2 × 10^7^ to 2 × 10^2^ colony forming units (CFU)/mL. All bacterial dilutions were boiled in a water bath for 10 min, and centrifuged at 10,000 rpm for 5 min. The supernatant containing the DNA from the lysed bacterial cells was collected and 5 μL of each dilution was used as template in the multiplex PCR assay.

### Chicken Farm *Salmonella* Isolates

Naturally contaminated samples were collected from feces and floors of a commercial chicken farm in Yangzhou, China according to the method ISO 6579-1:2017. The indigenous *Salmonella* strains were isolated as described (Cai et al., 2016; Zhou et al., 2017). In brief, all samples were cultured in buffered peptone water (50 mL; Difco, BD, Sparks, MD, United States) for pre-enrichment at 37°C for 24 h. The bacterial culture (0.1 mL) was subsequently inoculated into Rappaport-Vassiliadis R10 broth (10 mL; Difco, BD) and cultured at 42°C for 24 h. Then the bacteria broth was streaked on xylose lysine tergitol 4 agar plates (Difco, BD) and incubated continuously at 37°C for 20–24 h. The presumptive *Salmonella* colonies on all plates were individually collected and the biochemical tests were performed based on an API 20E assay system (BioMerieux, Marcy l’Etoile, France).

### Multiplex PCR Detection of the Chicken Farm *Salmonella* Isolates

To evaluate the diagnostic sensitivity of the multiplex PCR method on natural-occurring *Salmonella* isolates, the genomic DNA of *Salmonella* isolates from the chicken farm was extracted by the boiling method and used as PCR templates. The developed multiplex PCR results were then compared with the conventional *Salmonella* serotyping and biochemical tests.

### Traditional *Salmonella* Serotyping and Biochemical Testing

The serovars of the clinical *Salmonella* isolates were characterized using traditional *Salmonella* serotyping assays with diagnostic antisera for rapid agglutination following the White–Kauffmann–Le Minor instructions (Grimont and Weill, 2007). For differentiation of *S.* Gallinarum and *S.* Pullorum, dulcitol fermentation and ornithine decarboxylation tests were used to distinguish the two biovars.

## Results

### Bioinformatics Analysis and Primer Design

**FIGURE 1:**
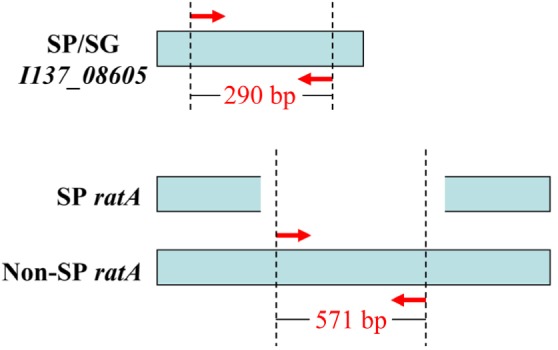
Primer design for the multiplex PCR method to distinguish *Salmonella enterica* serovar Gallinarum biovars Gallinarum (*S.* Gallinarum) and Pullorum (*S.* Pullorum) from other serovars. Gene *I137_08605* exists only in the two *Salmonella* biovars, and gene *ratA* of *S.* Pullorum has a region of difference (ROD) compared with that of other serovars, which were exploited to design the primers. The red arrows indicate the positions of the designed primers.

Bioinformatics analysis revealed that *I137_08605* existed only in *S.* Pullorum and *S.* Gallinarum, and was not present in any other *Salmonella* serovars, or in non-*Salmonella* strains that have 100% sequence similarity across the two *Salmonella* biovars that were included in the database (Supplementary Figure S1). The *ratA* gene of *S.* Pullorum was 4776 bp, covering 85% of the length of *ratA* in *S.* Gallinarum and other serovars. The truncated sequence was due to variations in the region of difference (ROD) of *ratA*, which was not found in any *S.* Pullorum. This truncation could be exploited to differentiate *S.* Pullorum from *S.* Gallinarum (Supplementary Figure S2). Therefore, two pairs of oligonucleotide primers that distinguished the two *Salmonella* biovars were designed, specifically targeting *I137_08605* and *ratA ROD* (**Table 1**). The primer sets in the multiplex PCR assay produced an amplicon of 290 bp specific to *I137_08605* for biovar Pullorum, and two amplicons of 290 and 571 bp specific to *I137_08605* and *ratA ROD*, respectively, for biovar Gallinarum (**Figure 1**).

### Analytical Selectivity of the Multiplex PCR Assay

**FIGURE 2:**
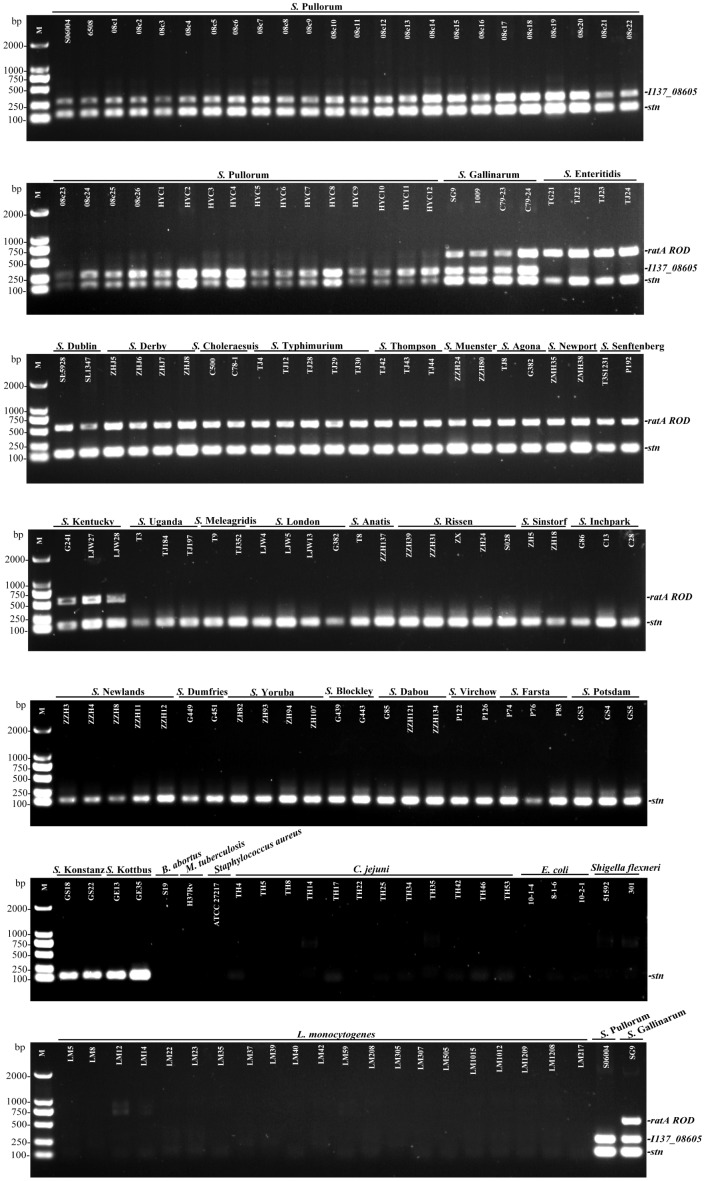
Selectivity of the multiplex PCR method for the identification of *S.* Pullorum and *S.* Gallinarum. The multiplex PCR assays, using genomic DNA from various *Salmonella* and non-*Salmonella* strains, were conducted using the designed primers targeting *stn* (131 bp), *I137_08605* (290 bp), and *ratA ROD* (571 bp). The three specific PCR products could be amplified in *S.* Gallinarum, while only *stn* and *I137_08605* genes could be amplified in *S.* Pullorum.

The multiplex PCR selectivity was evaluated by detecting the genomic DNA from 124 strains of various *Salmonella* serovars and 42 strains of different non-*Salmonella* pathogens. The multiplex PCR results showed that three specific bands corresponding to the amplification of *stn*, *I137_08605*, and *ratA ROD* from *S.* Gallinarum were generated, and only two specific bands corresponding to the amplification of *stn* and *I137_08605* was generated from *S.* Pullorum. In contrast, only two amplifications corresponding to *stn* and *ratA ROD* were produced in a portion of the other 29 *Salmonella* serovars. No amplification was detected for any of the non-*Salmonella* strains, suggesting that the developed multiplex PCR method had an excellent selectivity with 100% inclusivity and exclusivity. The relative specificity and relative sensitivity to discriminate negative and positive samples were both 100% for the multiplex PCR method (**Figure 2**).

### Analytical Detecting Limit of the Multiplex PCR Assay

The multiplex PCR sensitivity was evaluated by detecting serially diluted genomic DNA from *S.* Gallinarum and *S.* Pullorum. The results showed that *stn*, *I137_08605*, and *ratA ROD* could be amplified at the lowest concentration of 67.4 pg/μL, suggesting that at least 67.4 pg/μL of bacterial genomic DNA was required in order to identify and distinguish *S.* Gallinarum or *S.* Pullorum using this multiplex PCR method. In addition, the fewest number of *S.* Pullorum or *S.* Gallinarum cells the PCR method could detect was also determined. Based on the multiplex PCR assay on various concentrations of diluted *Salmonella* cells, we determined that the minimum number of bacteria able to be detected was 100 CFU (data not shown).

### Application of the Multiplex PCR Method

To evaluate the sensitivity of the multiplex PCR, additional *Salmonella* isolates were used for the method. The isolates of unknown serovars were collected from naturally contaminated samples from a chicken farm and were prepared for use as a template in order to evaluate the diagnostic efficiency of this multiplex PCR method. The PCR results showed that 21 samples generated only two specific band corresponding to amplification of *stn* and *I137_08605*, suggesting that the 21 isolates were *S.* Pullorum. Two samples from the chicken farm generated the three specific bands consistent with *stn*, *I137_08605*, and *ratA ROD* amplification, suggesting that these two isolated strains were *S.* Gallinarum (**Table 2**).

**Table 2:** *Salmonella* strains isolated from one chicken farm to examine the application of the developed multiplex PCR method.

Serovar (no. of isolates)	Isolate no.	PCR results (*ratA ROD*/*I137_08605*/*stn*)	Dulcitol fermentation	Ornithine decarboxylase
Pullorum (21)	Ch6	-/+/+	-	+
	Ch9	-/+/+	-	+
	Ch12	-/+/+	-	+
	Ch13	-/+/+	-	+
	Ch16	-/+/+	-	+
	Ch18	-/+/+	-	+
	Ch19	-/+/+	-	+
	Ch20	-/+/+	-	+
	Ch29	-/+/+	-	+
	Ch35	-/+/+	-	+
	Ch36	-/+/+	-	+
	Ch37	-/+/+	-	+
	Ch41	-/+/+	-	+
	Ch42	-/+/+	-	+
	Ch45	-/+/+	-	+
	Ch53	-/+/+	-	+
	Ch55	-/+/+	-	+
	Ch58	-/+/+	-	+
	Ch59	-/+/+	-	+
	Ch64	-/+/+	-	+
	Ch65	-/+/+	-	+
Gallinarum (2)	Ch33	+/+/+	+	-
	Ch47	+/+/+	+	-
Enteritidis (35)	Ch1	+/-/+	/	/
	Ch2	+/-/+	/	/
	Ch3	+/-/+	/	/
	Ch4	+/-/+	/	/
	Ch5	+/-/+	/	/
	Ch7	+/-/+	/	/
	Ch8	+/-/+	/	/
	Ch10	+/-/+	/	/
	Ch11	+/-/+	/	/
	Ch14	+/-/+	/	/
	Ch15	+/-/+	/	/
	Ch17	+/-/+	/	/
	Ch21	+/-/+	/	/
	Ch22	+/-/+	/	/
	Ch23	+/-/+	/	/
	Ch24	+/-/+	/	/
	Ch25	+/-/+	/	/
	Ch26	+/-/+	/	/
	Ch27	+/-/+	/	/
	Ch28	+/-/+	/	/
	Ch30	+/-/+	/	/
	Ch31	+/-/+	/	/
	Ch32	+/-/+	/	/
	Ch34	+/-/+	/	/
	Ch38	+/-/+	/	/
	Ch39	+/-/+	/	/
	Ch40	+/-/+	/	/
	Ch43	+/-/+	/	/
	Ch44	+/-/+	/	/
	Ch46	+/-/+	/	/
	Ch48	+/-/+	/	/
	Ch49	+/-/+	/	/
	Ch50	+/-/+	/	/
	Ch51	+/-/+	/	/
	Ch52	+/-/+	/	/
Indiana (13)	Ch54	-/-/+	/	/
	Ch56	-/-/+	/	/
	Ch57	-/-/+	/	/
	Ch60	-/-/+	/	/
	Ch61	-/-/+	/	/
	Ch62	-/-/+	/	/
	Ch63	-/-/+	/	/
	Ch66	-/-/+	/	/
	Ch67	-/-/+	/	/
	Ch68	-/-/+	/	/
	Ch69	-/-/+	/	/
	Ch70	-/-/+	/	/
	Ch71	-/-/+	/	/

### Traditional Serotyping and Biochemical Identification of the *Salmonella* Isolates

To validate the accuracy of the multiplex PCR assay, traditional *Salmonella* serotyping and biochemical assays were conducted on the *Salmonella* isolates of the chicken farm. The results showed that the 71 *Salmonella* isolates included 21 strains of *S.* Pullorum, two strains of *S.* Gallinarum, 13 strains of *S.* Indiana, and 35 strains of *S.* Enteritidis, which were completely concordant with the multiplex PCR results (**Table 2**).

## Discussion

*Salmonella* Pullorum and *S.* Gallinarum belong to important bacterial pathogens and have resulted in enormous economic losses in poultry, particularly in the developing countries. *S.* Pullorum infection of young birds often leads to a high mortality. Infected adult birds show decreased egg laying, weight loss, dysentery, and most importantly persistent infection occurs in most recovered birds, which may lead to horizontal and vertical transmission (Barrow and Freitas Neto, 2011). *S.* Gallinarum can infect birds of any age and is highly pathogenic, causing fowl typhoid, which usually results in systemic infection, and may cause 40–80% mortality in the flock (Ribeiro et al., 2009). Although different diseases are caused by these two pathogens, they are genetically and phenotypically alike (Batista et al., 2015).

Definitive diagnosis and timely removal of infected birds are crucial to prevent the spread and control the prevalence of *S.* Gallinarum and *S.* Pullorum in poultry. Currently, the determination of *Salmonella* serotype is mainly based on the White–Kauffmann–Le Minor scheme by identifying the somatic (O) and flagellar (H) antigens, and some special biochemical tests (Grimont and Weill, 2007) according to ISO 6579-1:2017. However, it is difficult to differentiate between *S.* Gallinarum and *S.* Pullorum since they share the same 1, 9, and 12 O antigens (Christensen et al., 1993). Therefore, development of a rapid and reliable method for the accurate identification and discrimination of biovars Gallinarum and Pullorum would allow for earlier confirmation of the pathogens and subsequently a more effective elimination of the diseases from flocks.

Because *Salmonella* biovars Pullorum and Gallinarum belong to the same serovar, but different biovars, their identification and differentiation is primarily based on biochemical reactions. Dulcitol fermentation and ornithine decarboxylation are currently the most widely used methods, and are officially recognized. However, these traditional methods have inherited drawbacks, such as time-consumption, labor-intension, and costliness, especially when many samples must be analyzed in a short time. Thus, an affordable and rapid diagnosis method is urgently desired for the identification of *S.* Gallinarum and *S.* Pullorum.

Molecular assays have been shown to be highly specific and sensitive for identifying causative pathogens of infectious diseases, and for differentiating closely related bacteria (Hu et al., 2006). Many studies have shown that PCR-based detection of *Salmonella* serovars is more sensitive, easier, and faster than traditional microbiologic methods (Oliveira et al., 2002; Soria et al., 2012). Therefore, a novel multiplex PCR assay based on three specific genes, *stn*, *I137_08605*, and *ratA ROD*, was developed for the identification and differentiation of *Salmonella* serovar Gallinarum biovars Gallinarum and Pullorum. The unique gene *I137_08605*, present only in biovars Gallinarum and Pullorum, was a common feature shared by these biovars, but was not present in any other known *Salmonella* serovars or species. Sequence analysis of *ratA ROD* in serovar Gallinarum strains revealed a deletion in biovar Pullorum. The multiplex PCR developed in this study had 100% specificity, and the lower limit of DNA and the fewest number of bacteria that could be detected were 67.4 pg/μL and 100 CFU, respectively, which were comparable with our previous study (Xiong et al., 2017). Our results showed that the combined amplification of *stn*, *I137_08605*, and *ratA ROD* could be used to identify, as well as to distinguish, *S.* Gallinarum and *S.* Pullorum rapidly and reliably. This newly developed multiplex PCR assay may be applicable to epidemiological investigations and will be a potent diagnostic method in veterinary clinical laboratories.

## Conclusion

In summary, we developed a simple, rapid, accurate, and cost-effective multiplex PCR assay to identify and discriminate *S.* Pullorum and *S.* Gallinarum from other serovars and non-*Salmonella* strains. The multiplex PCR method was based on the three genes *stn*, *I137_08605*, and *ratA ROD*, and it has excellent selectivity and potent sensitivity. The method was applied to detect a group of naturally occurring *Salmonella* isolates, thereby confirming its efficiency in the commercial farm. This highly accurate, biovar-specific, multiplex PCR system will be useful for the rapid differential diagnosis of biovars Pullorum and Gallinarum in veterinary laboratories. This assay could contribute to earlier confirmation of pathogens, and allow for more effective elimination of the diseases from flocks.

## Author Contributions

ZP and XJ designed the experiments. DX and LS designed the experiments, performed the PCR assays, and analyzed the results. ZP, XJ, and DX wrote the paper. All authors read and approved the final manuscript.

## Conflict of Interest Statement

The authors declare that the research was conducted in the absence of any commercial or financial relationships that could be construed as a potential conflict of interest.
