# Supplementary material for: Identification and Discrimination of Salmonella enterica Serovar Gallinarum Biovars Pullorum and Gallinarum Based on a One-Step Multiplex PCR Assay
Source: Front Microbiol. 2018 Jul 31;9:1718. doi: 10.3389/fmicb.2018.01718 (PMC6079294; doi:10.3389/fmicb.2018.01718)
Supplement: Supplementary file 1 [file Image_1.PDF]

BLAST Results

Job title: I137_08605

**RID**	97D6VP4H01R (Expires on 02-27 14:18 pm)
**Query ID**	lcl|Query_91429
**Description**	I137_08605
**Molecule type**	nucleic acid
**Query Length**	405
**Database Name**	nr
**Description**	Nucleotide collection (nt)
**Program**	BLASTN 2.8.0+

Graphic Summary

Distribution of the top 9 Blast Hits on 9 subject sequences

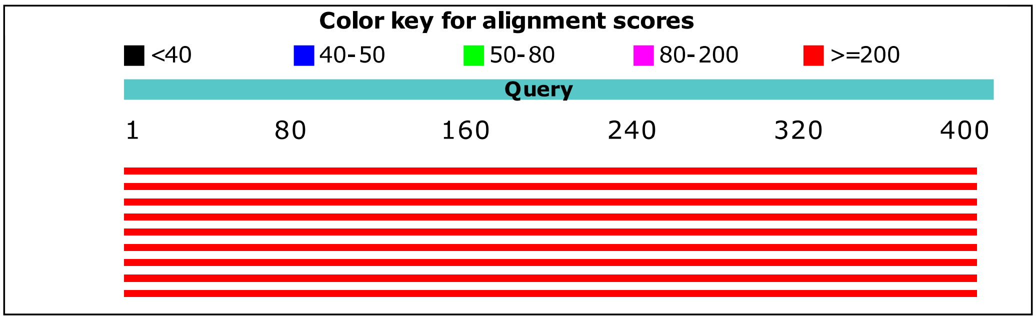

Descriptions

Sequences producing significant alignments:

Description	Max score	Total score	Query cover	E value	Ident	Accession
Salmonella enterica subsp. enterica serovar Gallinarum str. 9184, complete genome	749	749	100%	0.0	100%	CP019035.1
Salmonella enterica subsp. enterica serovar Pullorum str. ATCC 9120, complete genome	749	749	100%	0.0	100%	CP012347.1
Salmonella enterica subsp. enterica serovar Pullorum genome assembly S44987_1, chromosome : I	749	749	100%	0.0	100%	LK931482.1
Salmonella enterica subsp. enterica serovar Gallinarum/pullorum str. CDC1983-67, complete genome	749	749	100%	0.0	100%	CP003786.1
Salmonella enterica subsp. enterica serovar Pullorum str. S06004, complete genome	749	749	100%	0.0	100%	CP006575.1
Salmonella enterica subsp. enterica serovar Gallinarum/pullorum str. RKS5078, complete genome	749	749	100%	0.0	100%	CP003047.1
Salmonella enterica subsp. enterica serovar Gallinarum clone USG3f-USG3r RHS-family protein (rhs) pseudogene, partial sequence; and conserved hypothetical protein gene, complete cds	749	749	100%	0.0	100%	HQ014666.1
Salmonella enterica subsp. enterica serovar Gallinarum clone USG4f-USG3r RHS-family protein (rhs) pseudogene, partial sequence; and conserved hypothetical protein gene, complete cds	749	749	100%	0.0	100%	HQ014667.1
Salmonella enterica subsp. enterica serovar Gallinarum str. 287/91 complete genome	749	749	100%	0.0	100%	AM933173.1

Alignments

Salmonella enterica subsp. enterica serovar Gallinarum str. 9184, complete genome

Sequence ID: **CP019035.1** Length: 4609911 Number of Matches: 1

Range 1: 3028135 to 3028539

Score	Expect	Identities	Gaps	Strand	Frame
749 bits(405)	0.0()	405/405(100%)	0/405(0%)	Plus/Plus	

Features:

```
Query  1        TTATTTAGAACAGTTAGATATCACTGGAGACTCTGAGGACAAAACATCAATGATATCATT  60
                ||||||||||||||||||||||||||||||||||||||||||||||||||||||||||||
Sbjct  3028135  TTATTTAGAACAGTTAGATATCACTGGAGACTCTGAGGACAAAACATCAATGATATCATT  3028194

Query  61       TACTGTCACAATCAAATAATGAATAATTTTCTCTTTTTCATGTATATTAAAACTCTGGTC  120
                ||||||||||||||||||||||||||||||||||||||||||||||||||||||||||||
Sbjct  3028195  TACTGTCACAATCAAATAATGAATAATTTTCTCTTTTTCATGTATATTAAAACTCTGGTC  3028254

Query  121      ATTAAACCATTCCAGATAAGATGAGTCCTCAACCTTATAAATACCTACTCGCATTTTTCC  180
                ||||||||||||||||||||||||||||||||||||||||||||||||||||||||||||
Sbjct  3028255  ATTAAACCATTCCAGATAAGATGAGTCCTCAACCTTATAAATACCTACTCGCATTTTTCC  3028314

Query  181      TTTTTGCTCACCCAACATTTTCAACAGATCACCTTCATCAGTGACACGAAATGAATGAGT  240
                ||||||||||||||||||||||||||||||||||||||||||||||||||||||||||||
Sbjct  3028315  TTTTTGCTCACCCAACATTTTCAACAGATCACCTTCATCAGTGACACGAAATGAATGAGT  3028374

Query  241      CCAATCaaaaaaaaaTCGAAAATCCGAATTCACTCCATCAAGAGTAACTATAATCTCAAG  300
                ||||||||||||||||||||||||||||||||||||||||||||||||||||||||||||
Sbjct  3028375  CCAATCAAAAAAAAATCGAAAATCCGAATTCACTCCATCAAGAGTAACTATAATCTCAAG  3028434

Query  301      ACTCCCTGCCCTATAATGAAAATCAATTATCTGACTACCTTTTAAAGATATATTTTGGGG  360
                ||||||||||||||||||||||||||||||||||||||||||||||||||||||||||||
Sbjct  3028435  ACTCCCTGCCCTATAATGAAAATCAATTATCTGACTACCTTTTAAAGATATATTTTGGGG  3028494

Query  361      GGTAATATCAACCAATTTTTCAATCATATATTCCTCACTAATCAT  405
                |||||||||||||||||||||||||||||||||||||||||||||
Sbjct  3028495  GGTAATATCAACCAATTTTTCAATCATATATTCCTCACTAATCAT  3028539
```

Salmonella enterica subsp. enterica serovar Pullorum str. ATCC 9120, complete genome
Sequence ID: **CP012347.1** Length: 4694842 Number of Matches: 1
Range 1: 2764607 to 2765011

Score	Expect	Identities	Gaps	Strand	Frame
749 bits(405)	0.0()	405/405(100%)	0/405(0%)	Plus/Plus	

Features:

```
Query  1        TTATTTAGAACAGTTAGATATCACTGGAGACTCTGAGGACAAAACATCAATGATATCATT  60
                ||||||||||||||||||||||||||||||||||||||||||||||||||||||||||||
Sbjct  2764607  TTATTTAGAACAGTTAGATATCACTGGAGACTCTGAGGACAAAACATCAATGATATCATT  2764666
Query  61       TACTGTCACAATCAAATAATGAATAATTTTCTCTTTTTCATGTATATTAAAACTCTGGTC  120
                ||||||||||||||||||||||||||||||||||||||||||||||||||||||||||||
Sbjct  2764667  TACTGTCACAATCAAATAATGAATAATTTTCTCTTTTTCATGTATATTAAAACTCTGGTC  2764726
Query  121      ATTAAACCATTCCAGATAAGATGAGTCCTCAACCTTATAAATACCTACTCGCATTTTTCC  180
                ||||||||||||||||||||||||||||||||||||||||||||||||||||||||||||
Sbjct  2764727  ATTAAACCATTCCAGATAAGATGAGTCCTCAACCTTATAAATACCTACTCGCATTTTTCC  2764786
Query  181      TTTTTGCTCACCCAACATTTTCAACAGATCACCTTCATCAGTGACACGAAATGAATGAGT  240
                ||||||||||||||||||||||||||||||||||||||||||||||||||||||||||||
Sbjct  2764787  TTTTTGCTCACCCAACATTTTCAACAGATCACCTTCATCAGTGACACGAAATGAATGAGT  2764846
Query  241      CCAATCaaaaaaaaaTCGAAAATCCGAATTCACTCCATCAAGAGTAACTATAATCTCAAG  300
                ||||||||||||||||||||||||||||||||||||||||||||||||||||||||||||
Sbjct  2764847  CCAATCAAAAAAAAATCGAAAATCCGAATTCACTCCATCAAGAGTAACTATAATCTCAAG  2764906
Query  301      ACTCCCTGCCCTATAATGAAAATCAATTATCTGACTACCTTTTAAAGATATATTTTGGGG  360
                ||||||||||||||||||||||||||||||||||||||||||||||||||||||||||||
Sbjct  2764907  ACTCCCTGCCCTATAATGAAAATCAATTATCTGACTACCTTTTAAAGATATATTTTGGGG  2764966
Query  361      GGTAATATCAACCAATTTTTCAATCATATATTCCTCACTAATCAT  405
                |||||||||||||||||||||||||||||||||||||||||||||
Sbjct  2764967  GGTAATATCAACCAATTTTTCAATCATATATTCCTCACTAATCAT  2765011
```

Salmonella enterica subsp. enterica serovar Pullorum genome assembly S44987_1, chromosome : I
Sequence ID: **LK931482.1** Length: 4620579 Number of Matches: 1
Range 1: 1065817 to 1066221

Score	Expect	Identities	Gaps	Strand	Frame
749 bits(405)	0.0()	405/405(100%)	0/405(0%)	Plus/Minus	

Features:

```
Query  1        TTATTTAGAACAGTTAGATATCACTGGAGACTCTGAGGACAAAACATCAATGATATCATT  60
                ||||||||||||||||||||||||||||||||||||||||||||||||||||||||||||
Sbjct  1066221  TTATTTAGAACAGTTAGATATCACTGGAGACTCTGAGGACAAAACATCAATGATATCATT  1066162
Query  61       TACTGTCACAATCAAATAATGAATAATTTTCTCTTTTTCATGTATATTAAAACTCTGGTC  120
                ||||||||||||||||||||||||||||||||||||||||||||||||||||||||||||
Sbjct  1066161  TACTGTCACAATCAAATAATGAATAATTTTCTCTTTTTCATGTATATTAAAACTCTGGTC  1066102
Query  121      ATTAAACCATTCCAGATAAGATGAGTCCTCAACCTTATAAATACCTACTCGCATTTTTCC  180
                ||||||||||||||||||||||||||||||||||||||||||||||||||||||||||||
Sbjct  1066101  ATTAAACCATTCCAGATAAGATGAGTCCTCAACCTTATAAATACCTACTCGCATTTTTCC  1066042
Query  181      TTTTTGCTCACCCAACATTTTCAACAGATCACCTTCATCAGTGACACGAAATGAATGAGT  240
                ||||||||||||||||||||||||||||||||||||||||||||||||||||||||||||
Sbjct  1066041  TTTTTGCTCACCCAACATTTTCAACAGATCACCTTCATCAGTGACACGAAATGAATGAGT  1065982
Query  241      CCAATCaaaaaaaaaTCGAAAATCCGAATTCACTCCATCAAGAGTAACTATAATCTCAAG  300
                ||||||||||||||||||||||||||||||||||||||||||||||||||||||||||||
Sbjct  1065981  CCAATCAAAAAAAAATCGAAAATCCGAATTCACTCCATCAAGAGTAACTATAATCTCAAG  1065922
Query  301      ACTCCCTGCCCTATAATGAAAATCAATTATCTGACTACCTTTTAAAGATATATTTTGGGG  360
                ||||||||||||||||||||||||||||||||||||||||||||||||||||||||||||
Sbjct  1065921  ACTCCCTGCCCTATAATGAAAATCAATTATCTGACTACCTTTTAAAGATATATTTTGGGG  1065862
Query  361      GGTAATATCAACCAATTTTTCAATCATATATTCCTCACTAATCAT  405
                |||||||||||||||||||||||||||||||||||||||||||||
Sbjct  1065861  GGTAATATCAACCAATTTTTCAATCATATATTCCTCACTAATCAT  1065817
```

Salmonella enterica subsp. enterica serovar Gallinarum/pullorum str. CDC1983-67, complete genome
Sequence ID: **CP003786.1** Length: 4623089 Number of Matches: 1
Range 1: 1874619 to 1875023

Score	Expect	Identities	Gaps	Strand	Frame
749 bits(405)	0.0()	405/405(100%)	0/405(0%)	Plus/Plus	

Features:

```
Query  1        TTATTTAGAACAGTTAGATATCACTGGAGACTCTGAGGACAAAACATCAATGATATCATT  60
                ||||||||||||||||||||||||||||||||||||||||||||||||||||||||||||
Sbjct  1874619  TTATTTAGAACAGTTAGATATCACTGGAGACTCTGAGGACAAAACATCAATGATATCATT  1874678
Query  61       TACTGTCACAATCAAATAATGAATAATTTTCTCTTTTTCATGTATATTAAAACTCTGGTC  120
                ||||||||||||||||||||||||||||||||||||||||||||||||||||||||||||
Sbjct  1874679  TACTGTCACAATCAAATAATGAATAATTTTCTCTTTTTCATGTATATTAAAACTCTGGTC  1874738
Query  121      ATTAAACCATTCCAGATAAGATGAGTCCTCAACCTTATAAATACCTACTCGCATTTTTCC  180
```

Supplementary FIGURE S1 | BLAST search results using *Salmonella* Pullorum *I137_08605* nucleotide sequence (GenBank accession no. CP006575.1 segment 1847386-1847790) against the nucleotide collection (nr/nt) database. The maximum number of aligned sequences to display was set to the maximum value of 20,000, and the other parameters were set to default values. The results showed that *I137_08605* gene is present only in *S.* Gallinarum and *S.* Pullorum.
