# Supplementary material for: Identification and Discrimination of Salmonella enterica Serovar Gallinarum Biovars Pullorum and Gallinarum Based on a One-Step Multiplex PCR Assay
Source: Front Microbiol. 2018 Jul 31;9:1718. doi: 10.3389/fmicb.2018.01718 (PMC6079294; doi:10.3389/fmicb.2018.01718)
Supplement: Supplementary file 2 [file Image_2.PDF]

BLAST Results

Job title: ratA ROD

**RID**	97FFDK6K014 (Expires on 02-27 14:57 pm)
**Query ID**	lcl|Query_78461
**Description**	ratA ROD
**Molecule type**	nucleic acid
**Query Length**	790
**Database Name**	nr
**Description**	Nucleotide collection (nt)
**Program**	BLASTN 2.8.0+

Graphic Summary

Distribution of the top 100 Blast Hits on 526 subject sequences

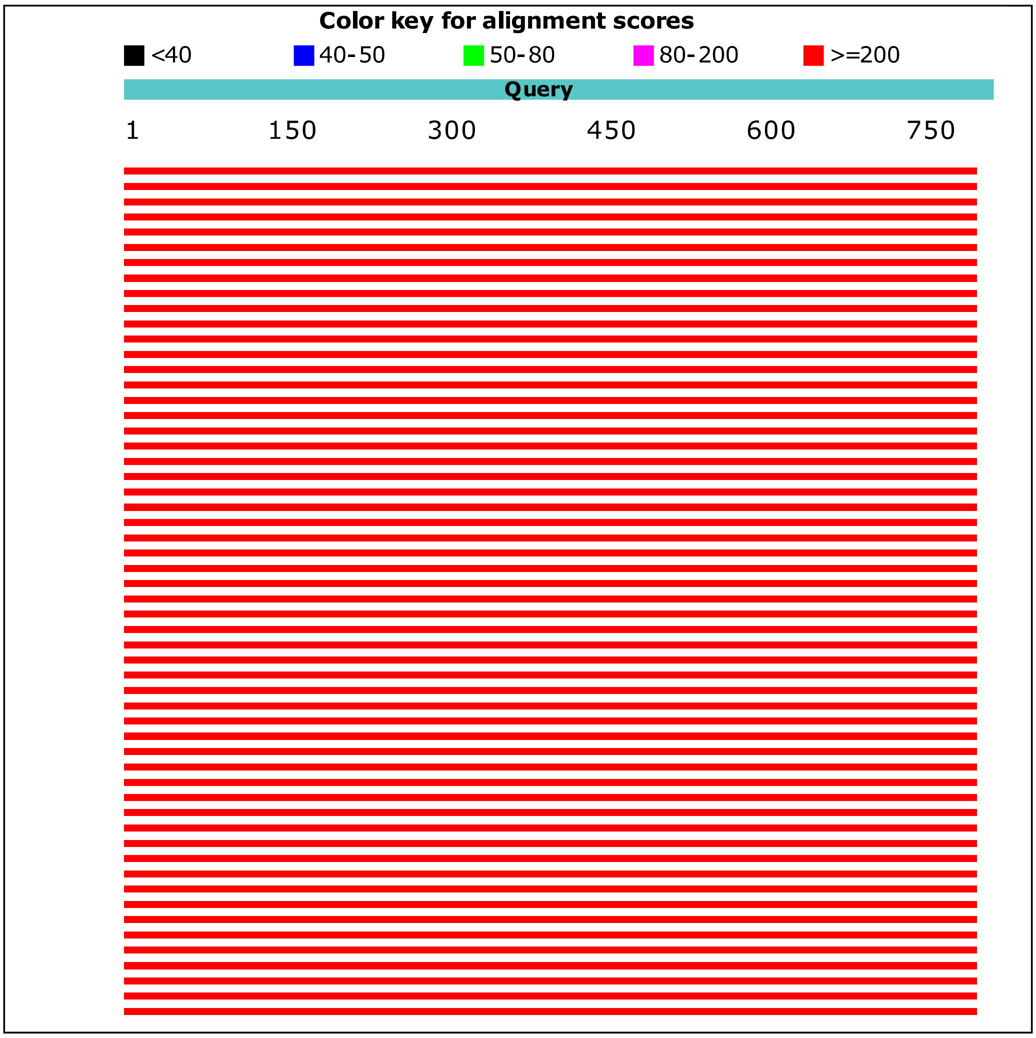

Descriptions

Sequences producing significant alignments:

Description	Max score	Total score	Query cover	E value	Ident	Accession
Salmonella enterica strain MFDS1004839 chromosome, complete genome	1459	1459	100%	0.0	100%	CP026569.1
Salmonella enterica strain FDAARGOS_70 chromosome, complete genome	1459	1459	100%	0.0	100%	CP026052.1
Salmonella enterica subsp. enterica serovar Typhimurium strain FORC50 chromosome, complete genome	1459	1459	100%	0.0	100%	CP019383.1
Salmonella enterica subsp. enterica serovar Enteritidis strain SJTUF10984 chromosome, complete genome	1459	1459	100%	0.0	100%	CP015526.1
Salmonella enterica subsp. enterica serovar Enteritidis strain SJTUF10978 chromosome, complete genome	1459	1459	100%	0.0	100%	CP015524.1
Salmonella enterica subsp. enterica serovar Enteritidis strain 95-0621 chromosome, complete genome	1459	1459	100%	0.0	100%	CP018661.1
Salmonella enterica subsp. enterica serovar Enteritidis strain 93-0639 chromosome, complete genome	1459	1459	100%	0.0	100%	CP018659.1
Salmonella enterica subsp. enterica serovar Enteritidis strain 81-1706 chromosome, complete genome	1459	1459	100%	0.0	100%	CP018655.1
Salmonella enterica subsp. enterica serovar Enteritidis strain 81-1705 chromosome, complete genome	1459	1459	100%	0.0	100%	CP018651.1
Salmonella enterica subsp. enterica serovar Enteritidis strain 81-1607 chromosome, complete genome	1459	1459	100%	0.0	100%	CP018648.1
Salmonella enterica subsp. enterica serovar Enteritidis strain 81-1435, complete genome	1459	1459	100%	0.0	100%	CP018647.1
Salmonella enterica subsp. enterica serovar Enteritidis strain 79-2359 chromosome, complete genome	1459	1459	100%	0.0	100%	CP018645.1
Salmonella enterica subsp. enterica serovar Enteritidis strain 77-2980, complete genome	1459	1459	100%	0.0	100%	CP018644.1
Salmonella enterica subsp. enterica serovar Enteritidis strain 70-1605, complete sequence	1459	1459	100%	0.0	100%	CP018640.1
Salmonella enterica subsp. enterica serovar Enteritidis strain 69-3861, complete genome	1459	1459	100%	0.0	100%	CP018637.1
Salmonella enterica subsp. enterica serovar Enteritidis strain 56-3991, complete genome	1459	1459	100%	0.0	100%	CP018635.1
Salmonella enterica subsp. enterica serovar Enteritidis strain 49-2444 chromosome, complete genome	1459	1459	100%	0.0	100%	CP018633.1
Salmonella enterica subsp. enterica strain RM11060 chromosome, complete genome	1459	1459	100%	0.0	100%	CP022658.1
Salmonella enterica strain FORC_051 chromosome, complete genome	1459	1459	100%	0.0	100%	CP017232.1
Salmonella enterica strain FORC_056 chromosome, complete genome	1459	1459	100%	0.0	100%	CP017177.1
Salmonella enterica subsp. enterica serovar Enteritidis strain FORC_052, complete genome	1459	1459	100%	0.0	100%	CP016754.1
Salmonella enterica subsp. enterica serovar Enteritidis strain CFSAN051873, complete	1459	1459	100%	0.0	100%	CP022003.1

genome						
Salmonella enterica subsp. enterica strain CFSAN033543, complete genome	1459	1459	100%	0.0	100%	CP020825.1
Salmonella enterica subsp. enterica strain CFSAN033541, complete genome	1459	1459	100%	0.0	100%	CP020823.1
Salmonella enterica subsp. enterica serovar Wandsworth str. SA20092095, complete genome	1459	1459	100%	0.0	100%	CP019417.1
Salmonella enterica subsp. enterica serovar Moscow str. S-1843, complete genome	1459	1459	100%	0.0	100%	CP019415.1
Salmonella enterica subsp. enterica serovar Blegdam str. S-1824, complete genome	1459	1459	100%	0.0	100%	CP019406.1
Salmonella enterica subsp. enterica serovar Gallinarum str. 9184, complete genome	1459	1459	100%	0.0	100%	CP019035.1
Salmonella enterica subsp. enterica serovar Newport strain 0007-33, complete genome	1459	1459	100%	0.0	100%	CP013685.1
Salmonella enterica subsp. enterica serovar Newport strain CFSAN003890, complete genome	1459	1459	100%	0.0	100%	CP016012.1
Salmonella enterica subsp. enterica serovar Newport str. WA_14882, complete genome	1459	1459	100%	0.0	100%	CP016357.1
Salmonella enterica subsp. enterica serovar Newport strain 0307-213, complete genome	1459	1459	100%	0.0	100%	CP012599.1
Salmonella enterica subsp. enterica serovar Newport strain 0211-109, complete genome	1459	1459	100%	0.0	100%	CP012598.1
Salmonella enterica subsp. enterica serovar Newport strain 0112-791, complete genome	1459	1459	100%	0.0	100%	CP012597.1
Salmonella enterica strain FORC_019, complete genome	1459	1459	100%	0.0	100%	CP012396.1
Salmonella enterica subsp. enterica serovar Enteritidis strain OLF-00D989 87-1, complete genome	1459	1459	100%	0.0	100%	CP011942.1
Salmonella enterica subsp. enterica serovar Enteritidis str. SA20094177 genome	1459	1459	100%	0.0	100%	CP007468.2
Salmonella enterica subsp. enterica serovar Enteritidis str. EC20110222 genome	1459	1459	100%	0.0	100%	CP007323.2
Salmonella enterica subsp. enterica serovar Enteritidis str. EC20120685 genome	1459	1459	100%	0.0	100%	CP007339.2
Salmonella enterica subsp. enterica serovar Enteritidis str. EC20120213 genome	1459	1459	100%	0.0	100%	CP007344.2
Salmonella enterica subsp. enterica serovar Enteritidis str. EC20120968 genome	1459	1459	100%	0.0	100%	CP007378.2
Salmonella enterica subsp. enterica serovar Enteritidis str. SA20082034, complete genome	1459	1459	100%	0.0	100%	CP007425.2
Salmonella enterica subsp. enterica serovar Enteritidis str. EC20111515 genome	1459	1459	100%	0.0	100%	CP007325.2
Salmonella enterica subsp. enterica serovar Enteritidis str. EC20111510 genome	1459	1459	100%	0.0	100%	CP007498.2
Salmonella enterica subsp. enterica serovar Enteritidis str. SA20094301 genome	1459	1459	100%	0.0	100%	CP007469.2
Salmonella enterica subsp. enterica serovar Enteritidis str. SA20084824 genome	1459	1459	100%	0.0	100%	CP007467.2
Salmonella enterica subsp. enterica serovar Enteritidis str. SA20084644 genome	1459	1459	100%	0.0	100%	CP007466.2
Salmonella enterica subsp. enterica serovar Enteritidis str. EC20121747 genome	1459	1459	100%	0.0	100%	CP007464.2
Salmonella enterica subsp. enterica serovar Enteritidis str. EC20120929 genome	1459	1459	100%	0.0	100%	CP007463.2
Salmonella enterica subsp. enterica serovar Enteritidis str. EC20120009 genome	1459	1459	100%	0.0	100%	CP007438.2
Salmonella enterica subsp. enterica serovar Enteritidis str. EC20120051 genome	1459	1459	100%	0.0	100%	CP007433.2
Salmonella enterica subsp. enterica serovar Enteritidis str. EC20121765 genome	1459	1459	100%	0.0	100%	CP007429.2

Salmonella enterica subsp. enterica serovar Enteritidis str. EC20120677 genome	1459	1459	100%	0.0	100%	CP007428.2
Salmonella enterica subsp. enterica serovar Enteritidis str. SA20100239 genome	1459	1459	100%	0.0	100%	CP007427.2
Salmonella enterica subsp. enterica serovar Enteritidis str. EC20100131 genome	1459	1459	100%	0.0	100%	CP007432.2
Salmonella enterica subsp. enterica serovar Enteritidis str. SA20094682 genome	1459	1459	100%	0.0	100%	CP007431.2
Salmonella enterica subsp. enterica serovar Enteritidis str. EC20090195 genome	1459	1459	100%	0.0	100%	CP007430.2
Salmonella enterica subsp. enterica serovar Enteritidis str. SA20085285, complete genome	1459	1459	100%	0.0	100%	CP007426.2
Salmonella enterica subsp. enterica serovar Enteritidis str. EC20120774 genome	1459	1459	100%	0.0	100%	CP007404.2
Salmonella enterica subsp. enterica serovar Enteritidis str. EC20121542 genome	1459	1459	100%	0.0	100%	CP007368.2
Salmonella enterica subsp. enterica serovar Enteritidis str. EC20121541 genome	1459	1459	100%	0.0	100%	CP007367.2
Salmonella enterica subsp. enterica serovar Enteritidis str. EC20121004 genome	1459	1459	100%	0.0	100%	CP007366.2
Salmonella enterica subsp. enterica serovar Enteritidis str. EC20121744 genome	1459	1459	100%	0.0	100%	CP007373.2
Salmonella enterica subsp. enterica serovar Enteritidis str. SA20121703 genome	1459	1459	100%	0.0	100%	CP007372.2
Salmonella enterica subsp. enterica serovar Enteritidis str. EC20121689 genome	1459	1459	100%	0.0	100%	CP007371.2
Salmonella enterica subsp. enterica serovar Enteritidis str. EC20121671 genome	1459	1459	100%	0.0	100%	CP007369.2
Salmonella enterica subsp. enterica serovar Enteritidis str. EC20090531 genome	1459	1459	100%	0.0	100%	CP007422.2
Salmonella enterica subsp. enterica serovar Enteritidis str. EC20090884 genome	1459	1459	100%	0.0	100%	CP007421.2
Salmonella enterica subsp. enterica serovar Enteritidis str. EC20100103 genome	1459	1459	100%	0.0	100%	CP007420.2
Salmonella enterica subsp. enterica serovar Enteritidis str. EC20120200, complete genome	1459	1459	100%	0.0	100%	CP007434.2
Salmonella enterica subsp. enterica serovar Enteritidis str. EC20130348 genome	1459	1459	100%	0.0	100%	CP007424.2
Salmonella enterica subsp. enterica serovar Enteritidis str. EC20130347 genome	1459	1459	100%	0.0	100%	CP007423.2
Salmonella enterica subsp. enterica serovar Enteritidis str. EC20130346 genome	1459	1459	100%	0.0	100%	CP007419.2
Salmonella enterica subsp. enterica serovar Enteritidis str. EC20130345 genome	1459	1459	100%	0.0	100%	CP007418.2
Salmonella enterica subsp. enterica serovar Enteritidis str. SA20123395 genome	1459	1459	100%	0.0	100%	CP007417.2
Salmonella enterica subsp. enterica serovar Enteritidis str. EC20122045 genome	1459	1459	100%	0.0	100%	CP007416.2
Salmonella enterica subsp. enterica serovar Enteritidis str. EC20122033 genome	1459	1459	100%	0.0	100%	CP007415.2
Salmonella enterica subsp. enterica serovar Enteritidis str. EC20120528 genome	1459	1459	100%	0.0	100%	CP007354.2
Salmonella enterica subsp. enterica serovar Enteritidis str. SA20100349 genome	1459	1459	100%	0.0	100%	CP007361.2
Salmonella enterica subsp. enterica serovar Enteritidis str. EC20100134 genome	1459	1459	100%	0.0	100%	CP007359.2
Salmonella enterica subsp. enterica serovar Enteritidis str. EC20100130 genome	1459	1459	100%	0.0	100%	CP007358.2
Salmonella enterica subsp. enterica serovar Enteritidis str. EC20100100 genome	1459	1459	100%	0.0	100%	CP007357.2
Salmonella enterica subsp. enterica serovar Enteritidis str. EC20121825 genome	1459	1459	100%	0.0	100%	CP007382.2
Salmonella enterica subsp. enterica serovar						

Enteritidis str. EC20121812 genome	1459	1459	100%	0.0	100%	CP007381.2
Salmonella enterica subsp. enterica serovar Enteritidis str. EC20120970 genome	1459	1459	100%	0.0	100%	CP007380.2
Salmonella enterica subsp. enterica serovar Enteritidis str. EC20120969 genome	1459	1459	100%	0.0	100%	CP007379.2
Salmonella enterica subsp. enterica serovar Enteritidis str. EC20120963 genome	1459	1459	100%	0.0	100%	CP007377.2
Salmonella enterica subsp. enterica serovar Enteritidis str. EC20120927 genome	1459	1459	100%	0.0	100%	CP007376.2
Salmonella enterica subsp. enterica serovar Enteritidis str. EC20120994 genome	1459	1459	100%	0.0	100%	CP007365.2
Salmonella enterica subsp. enterica serovar Enteritidis str. SA20094350 genome	1459	1459	100%	0.0	100%	CP007311.2
Salmonella enterica subsp. enterica serovar Enteritidis str. SA20094079 genome	1459	1459	100%	0.0	100%	CP007310.2
Salmonella enterica subsp. enterica serovar Enteritidis str. SA20093977 genome	1459	1459	100%	0.0	100%	CP007309.2
Salmonella enterica subsp. enterica serovar Enteritidis str. SA20093950 genome	1459	1459	100%	0.0	100%	CP007308.2
Salmonella enterica subsp. enterica serovar Enteritidis str. SA20093788 genome	1459	1459	100%	0.0	100%	CP007307.2
Salmonella enterica subsp. enterica serovar Enteritidis str. SA20092320 genome	1459	1459	100%	0.0	100%	CP007334.2
Salmonella enterica subsp. enterica serovar Enteritidis str. EC20121177, complete genome	1459	1459	100%	0.0	100%	CP007333.2
Salmonella enterica subsp. enterica serovar Enteritidis str. EC20120555 genome	1459	1459	100%	0.0	100%	CP007364.2
Salmonella enterica subsp. enterica serovar Enteritidis str. EC20120548 genome	1459	1459	100%	0.0	100%	CP007363.2
Salmonella enterica subsp. enterica serovar Enteritidis str. EC20120544 genome	1459	1459	100%	0.0	100%	CP007362.2
Salmonella enterica subsp. enterica serovar Enteritidis str. EC20121750 genome	1459	1459	100%	0.0	100%	CP007396.2
Salmonella enterica subsp. enterica serovar Enteritidis str. EC20121746 genome	1459	1459	100%	0.0	100%	CP007374.2
Salmonella enterica subsp. enterica serovar Enteritidis str. EC20120925 genome	1459	1459	100%	0.0	100%	CP007375.2
Salmonella enterica subsp. enterica serovar Enteritidis str. EC20120775 genome	1459	1459	100%	0.0	100%	CP007405.2
Salmonella enterica subsp. enterica serovar Enteritidis str. EC20100325 genome	1459	1459	100%	0.0	100%	CP007360.2
Salmonella enterica subsp. enterica serovar Enteritidis str. EC20120505 genome	1459	1459	100%	0.0	100%	CP007353.2
Salmonella enterica subsp. enterica serovar Enteritidis str. EC20120498 genome	1459	1459	100%	0.0	100%	CP007352.2
Salmonella enterica subsp. enterica serovar Enteritidis str. EC20120497 genome	1459	1459	100%	0.0	100%	CP007351.2
Salmonella enterica subsp. enterica serovar Enteritidis str. EC20120496 genome	1459	1459	100%	0.0	100%	CP007350.2
Salmonella enterica subsp. enterica serovar Enteritidis str. EC20120469 genome	1459	1459	100%	0.0	100%	CP007349.2
Salmonella enterica subsp. enterica serovar Enteritidis str. EC20120356 genome	1459	1459	100%	0.0	100%	CP007348.2
Salmonella enterica subsp. enterica serovar Enteritidis str. EC20120240 genome	1459	1459	100%	0.0	100%	CP007347.2
Salmonella enterica subsp. enterica serovar Enteritidis str. EC20120229 genome	1459	1459	100%	0.0	100%	CP007346.2
Salmonella enterica subsp. enterica serovar Enteritidis str. EC20120219 genome	1459	1459	100%	0.0	100%	CP007345.2
Salmonella enterica subsp. enterica serovar Enteritidis str. EC20120686 genome	1459	1459	100%	0.0	100%	CP007340.2
Salmonella enterica subsp. enterica serovar Enteritidis str. EC20120597 genome	1459	1459	100%	0.0	100%	CP007338.2

Salmonella enterica subsp. enterica serovar Enteritidis str. EC20120580 genome	1459	1459	100%	0.0	100%	CP007335.2
Salmonella enterica subsp. enterica serovar Enteritidis str. EC20111576 genome	1459	1459	100%	0.0	100%	CP007328.2
Salmonella enterica subsp. enterica serovar Enteritidis str. EC20111554 genome	1459	1459	100%	0.0	100%	CP007326.2
Salmonella enterica subsp. enterica serovar Enteritidis str. EC20111514 genome	1459	1459	100%	0.0	100%	CP007324.2
Salmonella enterica subsp. enterica serovar Enteritidis str. SA20095440, complete genome	1459	1459	100%	0.0	100%	CP007319.2
Salmonella enterica subsp. enterica serovar Enteritidis str. SA20094389 genome	1459	1459	100%	0.0	100%	CP007314.2
Salmonella enterica subsp. enterica serovar Enteritidis str. SA20094383 genome	1459	1459	100%	0.0	100%	CP007313.2
Salmonella enterica subsp. enterica serovar Enteritidis str. SA20094352 complete genome	1459	1459	100%	0.0	100%	CP007312.2
Salmonella enterica subsp. enterica serovar Enteritidis str. SA20093784 genome	1459	1459	100%	0.0	100%	CP007306.2
Salmonella enterica subsp. enterica serovar Enteritidis str. SA20093543 genome	1459	1459	100%	0.0	100%	CP007305.2
Salmonella enterica subsp. enterica serovar Enteritidis str. SA20093538 genome	1459	1459	100%	0.0	100%	CP007304.2
Salmonella enterica subsp. enterica serovar Enteritidis str. SA20093430 genome	1459	1459	100%	0.0	100%	CP007303.2
Salmonella enterica subsp. enterica serovar Enteritidis str. EC20100089 genome	1459	1459	100%	0.0	100%	CP007356.2
Salmonella enterica subsp. enterica serovar Enteritidis str. EC20100088 genome	1459	1459	100%	0.0	100%	CP007355.2
Salmonella enterica subsp. enterica serovar Enteritidis str. EC20090530 genome	1459	1459	100%	0.0	100%	CP007298.2
Salmonella enterica subsp. enterica serovar Enteritidis str. SA20090435 genome	1459	1459	100%	0.0	100%	CP007297.2
Salmonella enterica subsp. enterica serovar Enteritidis str. SA20090419 genome	1459	1459	100%	0.0	100%	CP007296.2
Salmonella enterica subsp. enterica serovar Enteritidis str. SA19981522, complete genome	1459	1459	100%	0.0	100%	CP007286.2
Salmonella enterica subsp. enterica serovar Enteritidis str. SA19980677, complete genome	1459	1459	100%	0.0	100%	CP007285.2
Salmonella enterica subsp. enterica serovar Enteritidis str. SA19970769, complete genome	1459	1459	100%	0.0	100%	CP007283.2
Salmonella enterica subsp. enterica serovar Enteritidis str. SA19992322 genome	1459	1459	100%	0.0	100%	CP007291.2
Salmonella enterica subsp. enterica serovar Enteritidis str. SA19983126 genome	1459	1459	100%	0.0	100%	CP007290.2
Salmonella enterica subsp. enterica serovar Enteritidis str. SA19982831 genome	1459	1459	100%	0.0	100%	CP007289.2
Salmonella enterica subsp. enterica serovar Enteritidis str. SA19981857, complete genome	1459	1459	100%	0.0	100%	CP007288.2
Salmonella enterica subsp. enterica serovar Enteritidis str. SA19930684 genome	1459	1459	100%	0.0	100%	CP007277.2
Salmonella enterica subsp. enterica serovar Enteritidis str. SA20084384 genome	1459	1459	100%	0.0	100%	CP007295.2
Salmonella enterica subsp. enterica serovar Enteritidis str. SA20083636, complete genome	1459	1459	100%	0.0	100%	CP007294.2
Salmonella enterica subsp. enterica serovar Enteritidis str. SA20083456 genome	1459	1459	100%	0.0	100%	CP007293.2
Salmonella enterica subsp. enterica serovar Enteritidis str. SA19994216, complete genome	1459	1459	100%	0.0	100%	CP007292.2

Salmonella enterica subsp. enterica serovar Enteritidis str. SA19970510, complete genome	1459	1459	100%	0.0	100%	CP007282.2
Salmonella enterica subsp. enterica serovar Enteritidis str. SA19961622 genome	1459	1459	100%	0.0	100%	CP007281.2
Salmonella enterica subsp. enterica serovar Enteritidis str. SA19960848, complete genome	1459	1459	100%	0.0	100%	CP007280.2
Salmonella enterica subsp. enterica serovar Enteritidis str. SA19943269 genome	1459	1459	100%	0.0	100%	CP007279.2
Salmonella enterica subsp. enterica serovar Enteritidis str. SA19942384 genome	1459	1459	100%	0.0	100%	CP007278.2
Salmonella enterica subsp. enterica serovar Enteritidis str. SA20091739 genome	1459	1459	100%	0.0	100%	CP007301.2
Salmonella enterica subsp. enterica serovar Enteritidis str. SA20090877 genome	1459	1459	100%	0.0	100%	CP007300.2
Salmonella enterica subsp. enterica serovar Enteritidis str. SA20093266 genome	1459	1459	100%	0.0	100%	CP007274.2
Salmonella enterica subsp. enterica serovar Enteritidis str. EC20121180 genome	1459	1459	100%	0.0	100%	CP007273.2
Salmonella enterica subsp. enterica serovar Enteritidis str. EC20121179 genome	1459	1459	100%	0.0	100%	CP007272.2
Salmonella enterica subsp. enterica serovar Enteritidis str. EC20121178, complete genome	1459	1459	100%	0.0	100%	CP007271.2
Salmonella enterica subsp. enterica serovar Enteritidis str. EC20121176 genome	1459	1459	100%	0.0	100%	CP007270.2
Salmonella enterica subsp. enterica serovar Enteritidis str. EC20122031 genome	1459	1459	100%	0.0	100%	CP007414.2
Salmonella enterica subsp. enterica serovar Enteritidis str. EC20122026 genome	1459	1459	100%	0.0	100%	CP007413.2
Salmonella enterica subsp. enterica serovar Enteritidis str. EC20121990 genome	1459	1459	100%	0.0	100%	CP007411.2
Salmonella enterica subsp. enterica serovar Enteritidis str. SA19971331 genome	1459	1459	100%	0.0	100%	CP007284.2
Salmonella enterica subsp. enterica serovar Enteritidis str. EC20110223 genome	1459	1459	100%	0.0	100%	CP007266.2
Salmonella enterica subsp. enterica serovar Enteritidis str. EC20120918 genome	1459	1459	100%	0.0	100%	CP007408.2
Salmonella enterica subsp. enterica serovar Enteritidis str. EC20120917 genome	1459	1459	100%	0.0	100%	CP007407.2
Salmonella enterica subsp. enterica serovar Enteritidis str. EC20120776 genome	1459	1459	100%	0.0	100%	CP007406.2
Salmonella enterica subsp. enterica serovar Enteritidis str. EC20120773 genome	1459	1459	100%	0.0	100%	CP007403.2
Salmonella enterica subsp. enterica serovar Enteritidis str. EC20120765 genome	1459	1459	100%	0.0	100%	CP007402.2
Salmonella enterica subsp. enterica serovar Enteritidis str. EC20120734 genome	1459	1459	100%	0.0	100%	CP007400.2
Salmonella enterica subsp. enterica serovar Enteritidis str. EC20121753 genome	1459	1459	100%	0.0	100%	CP007398.2
Salmonella enterica subsp. enterica serovar Enteritidis str. EC20121751 genome	1459	1459	100%	0.0	100%	CP007397.2
Salmonella enterica subsp. enterica serovar Enteritidis str. EC20121748 genome	1459	1459	100%	0.0	100%	CP007395.2
Salmonella enterica subsp. enterica serovar Enteritidis str. EC20121989 genome	1459	1459	100%	0.0	100%	CP007388.2
Salmonella enterica subsp. enterica serovar Enteritidis str. EC20121986 genome	1459	1459	100%	0.0	100%	CP007387.2
Salmonella enterica subsp. enterica serovar Enteritidis str. EC20121976 genome	1459	1459	100%	0.0	100%	CP007386.2
Salmonella enterica subsp. enterica serovar Enteritidis str. EC20121970 genome	1459	1459	100%	0.0	100%	CP007385.2
Salmonella enterica subsp. enterica serovar Enteritidis str. EC20121969 genome	1459	1459	100%	0.0	100%	CP007384.2

Salmonella enterica subsp. enterica serovar Enteritidis str. EC20121826 genome	1459	1459	100%	0.0	100%	CP007383.2
Salmonella enterica subsp. enterica serovar Enteritidis str. EC20120722 genome	1459	1459	100%	0.0	100%	CP007343.2
Salmonella enterica subsp. enterica serovar Enteritidis str. EC20120697 genome	1459	1459	100%	0.0	100%	CP007342.2
Salmonella enterica subsp. enterica serovar Enteritidis str. EC20120687 genome	1459	1459	100%	0.0	100%	CP007341.2
Salmonella enterica subsp. enterica serovar Enteritidis str. EC20120007 genome	1459	1459	100%	0.0	100%	CP007331.2
Salmonella enterica subsp. enterica serovar Enteritidis str. EC20120003 genome	1459	1459	100%	0.0	100%	CP007330.2
Salmonella enterica subsp. enterica serovar Enteritidis str. EC20111561 genome	1459	1459	100%	0.0	100%	CP007327.2
Salmonella enterica subsp. enterica serovar Enteritidis str. EC20090332 genome	1459	1459	100%	0.0	100%	CP007322.2
Salmonella enterica subsp. enterica serovar Enteritidis str. EC20090193 genome	1459	1459	100%	0.0	100%	CP007321.2
Salmonella enterica subsp. enterica serovar Enteritidis str. EC20090135 genome	1459	1459	100%	0.0	100%	CP007320.2
Salmonella enterica subsp. enterica serovar Enteritidis str. SA20095309 genome	1459	1459	100%	0.0	100%	CP007318.2
Salmonella enterica subsp. enterica serovar Enteritidis str. SA20094803 genome	1459	1459	100%	0.0	100%	CP007317.2
Salmonella enterica subsp. enterica serovar Enteritidis str. SA20094642 genome	1459	1459	100%	0.0	100%	CP007316.2
Salmonella enterica subsp. enterica serovar Enteritidis str. SA20094521 genome	1459	1459	100%	0.0	100%	CP007315.2
Salmonella enterica subsp. enterica serovar Enteritidis str. EC20120590 genome	1459	1459	100%	0.0	100%	CP007337.2
Salmonella enterica subsp. enterica serovar Enteritidis strain CMCC50041, complete genome	1459	1459	100%	0.0	100%	CP013097.1
Salmonella enterica subsp. enterica serovar Enteritidis strain FORC_007, complete genome	1459	1459	100%	0.0	100%	CP009768.1
Salmonella enterica subsp. enterica serovar Enteritidis strain SEE2, complete genome	1459	1459	100%	0.0	100%	CP011791.1
Salmonella enterica subsp. enterica serovar Enteritidis strain SEE1, complete genome	1459	1459	100%	0.0	100%	CP011790.1
Salmonella enterica subsp. enterica serovar Enteritidis str. EC20120005, complete genome	1459	1459	100%	0.0	100%	CP007267.2
Salmonella enterica subsp. enterica serovar Enteritidis str. EC20120002 genome	1459	1459	100%	0.0	100%	CP007329.2
Salmonella enterica subsp. enterica serovar Enteritidis str. EC20090641, complete genome	1459	1459	100%	0.0	100%	CP007249.2
Salmonella enterica subsp. enterica serovar Enteritidis str. 18569, complete genome	1459	1459	100%	0.0	100%	CP011394.1
Salmonella enterica subsp. enterica serovar Newport str. CVM 21550, complete genome	1459	1459	100%	0.0	100%	CP010283.1
Salmonella enterica subsp. enterica serovar Newport str. CVM 21538, complete genome	1459	1459	100%	0.0	100%	CP010282.1
Salmonella enterica subsp. enterica serovar Newport str. CVM 22513, complete genome	1459	1459	100%	0.0	100%	CP010281.1
Salmonella enterica subsp. enterica serovar Newport str. CVM 22425, complete genome	1459	1459	100%	0.0	100%	CP010279.1
Salmonella enterica subsp. enterica serovar Newport str. CVM N1543, complete genome	1459	1459	100%	0.0	100%	CP010284.1
Salmonella enterica subsp. enterica serovar Enteritidis strain OLF-SE2-98984-6, complete genome	1459	1459	100%	0.0	100%	CP009084.2
Salmonella enterica subsp. enterica serovar Enteritidis strain OLF-SE3-98983-4, complete genome	1459	1459	100%	0.0	100%	CP009085.2

Salmonella enterica subsp. enterica serovar Newport str. CVM 22462, complete genome	1459	1459	100%	0.0	100%	CP010280.1
Salmonella enterica subsp. enterica serovar Newport str. CVM N18486, complete genome	1459	1459	100%	0.0	100%	CP009561.1
Salmonella enterica subsp. enterica serovar Enteritidis strain SEJ, complete genome	1459	1459	100%	0.0	100%	CP008928.1
Salmonella enterica subsp. enterica serovar Enteritidis strain OLF-SE6-00219-16, complete genome	1459	1459	100%	0.0	100%	CP009088.1
Salmonella enterica subsp. enterica serovar Enteritidis strain OLF-SE1-1019-1, complete genome	1459	1459	100%	0.0	100%	CP009083.1
Salmonella enterica subsp. enterica serovar Enteritidis strain OLF-SE5-1104-2, complete genome	1459	1459	100%	0.0	100%	CP009087.1
Salmonella enterica subsp. enterica serovar Enteritidis strain OLF-SE4-0317-8, complete genome	1459	1459	100%	0.0	100%	CP009086.1
Salmonella enterica subsp. enterica serovar Enteritidis strain OLF-SE11-10058, complete genome	1459	1459	100%	0.0	100%	CP009093.1
Salmonella enterica subsp. enterica serovar Enteritidis strain OLF-SE10-10052, complete genome	1459	1459	100%	0.0	100%	CP009092.1
Salmonella enterica subsp. enterica serovar Enteritidis strain OLF-SE8-1021710, complete genome	1459	1459	100%	0.0	100%	CP009090.1
Salmonella enterica subsp. enterica serovar Enteritidis strain OLF-SE7-100819, complete genome	1459	1459	100%	0.0	100%	CP009089.1
Salmonella enterica subsp. enterica serovar Enteritidis str. 77-1427, complete genome	1459	1459	100%	0.0	100%	CP007598.1
Salmonella enterica subsp. enterica serovar Enteritidis str. CDC_2010K_0968, complete genome	1459	1459	100%	0.0	100%	CP007528.1
Salmonella enterica subsp. enterica serovar Enteritidis strain Durban, complete genome	1459	1459	100%	0.0	100%	CP007507.1
Salmonella enterica subsp. enterica serovar Enteritidis str. EC20090698, complete genome	1459	1459	100%	0.0	100%	CP007248.1
Salmonella enterica subsp. enterica serovar Enteritidis str. EC20110221, complete genome	1459	1459	100%	0.0	100%	CP007247.1
Salmonella enterica subsp. enterica serovar Enteritidis str. EC20100101, complete genome	1459	1459	100%	0.0	100%	CP007246.1
Salmonella enterica subsp. enterica serovar Enteritidis str. EC20120008, complete genome	1459	1459	100%	0.0	100%	CP007245.1
Salmonella enterica subsp. enterica serovar Enteritidis str. EC20110354, complete genome	1459	1459	100%	0.0	100%	CP007175.1
Salmonella enterica subsp. enterica serovar Enteritidis str. EC20111174, complete genome	1459	1459	100%	0.0	100%	CP007253.1
Salmonella enterica subsp. enterica serovar Enteritidis str. EC20111175, complete genome	1459	1459	100%	0.0	100%	CP007252.1
Salmonella enterica subsp. enterica serovar Enteritidis str. EC20110353, complete genome	1459	1459	100%	0.0	100%	CP007251.1
Salmonella enterica subsp. enterica serovar Enteritidis str. EC20110355, complete genome	1459	1459	100%	0.0	100%	CP007250.1
Salmonella enterica subsp. enterica serovar Enteritidis str. SA19940857 genome	1459	1459	100%	0.0	100%	CP007465.1
Salmonella enterica subsp. enterica serovar						

Enteritidis str. EC20111095, complete genome	1459	1459	100%	0.0	100%	CP007254.1
Salmonella enterica subsp. enterica serovar Enteritidis str. EC20120916 genome	1459	1459	100%	0.0	100%	CP007332.1
Salmonella enterica subsp. enterica serovar Enteritidis str. EC20110356, complete genome	1459	1459	100%	0.0	100%	CP007262.1
Salmonella enterica subsp. enterica serovar Enteritidis str. EC20110357, complete genome	1459	1459	100%	0.0	100%	CP007261.1
Salmonella enterica subsp. enterica serovar Enteritidis str. EC20110358, complete genome	1459	1459	100%	0.0	100%	CP007260.1
Salmonella enterica subsp. enterica serovar Enteritidis str. EC20110359, complete genome	1459	1459	100%	0.0	100%	CP007259.1
Salmonella enterica subsp. enterica serovar Enteritidis str. EC20110360, complete genome	1459	1459	100%	0.0	100%	CP007258.1
Salmonella enterica subsp. enterica serovar Enteritidis str. EC20110361, complete genome	1459	1459	100%	0.0	100%	CP007263.1
Salmonella enterica subsp. enterica serovar Newport str. USMARC-S3124.1, complete genome	1459	1459	100%	0.0	100%	CP006631.1
Salmonella enterica subsp. enterica serovar Enteritidis str. P125109 complete genome	1459	1459	100%	0.0	100%	AM933172.1
Salmonella enterica subsp. enterica serovar Gallinarum str. 287/91 complete genome	1459	1459	100%	0.0	100%	AM933173.1
Salmonella enterica subsp. enterica serovar Newport str. SL254, complete genome	1459	1459	100%	0.0	100%	CP001113.1
Salmonella enterica subsp. enterica serovar Enteritidis str. EC20122022 genome	1456	1456	100%	0.0	99%	CP007412.2
Salmonella enterica subsp. enterica serovar Enteritidis str. EC20120738 genome	1456	1456	100%	0.0	99%	CP007401.2
Salmonella enterica subsp. enterica serovar Enteritidis str. EC20121672 genome	1456	1456	100%	0.0	99%	CP007370.2
Salmonella enterica subsp. enterica serovar Enteritidis str. EC20120581 genome	1456	1456	100%	0.0	99%	CP007336.2
Salmonella enterica subsp. enterica serovar Enteritidis str. SA20093421 genome	1456	1456	100%	0.0	99%	CP007302.2
Salmonella enterica subsp. enterica serovar Newport str. CVM 21554, complete genome	1454	1454	100%	0.0	99%	CP009565.1
Salmonella enterica subsp. enterica serovar Enteritidis strain OLF-SE9-10012, complete genome	1454	1454	100%	0.0	99%	CP009091.1
Salmonella enterica subsp. enterica serovar Nitra strain S-1687, complete genome	1448	1448	100%	0.0	99%	CP019416.1
Salmonella enterica subsp. enterica serovar Abony str. 0014, complete genome	1447	1447	99%	0.0	99%	CP007534.1
Salmonella enterica subsp. enterica strain FDAARGOS_54 chromosome, complete genome	1437	1437	100%	0.0	99%	CP026976.1
Salmonella enterica subsp. enterica serovar Crossness str. 1422-74, complete genome	1437	1437	100%	0.0	99%	CP019408.1
Salmonella enterica subsp. enterica serovar Heidelberg str. SARA35, complete genome	1437	1437	100%	0.0	99%	CP019176.1
Salmonella enterica subsp. enterica serovar Heidelberg strain SH13-004, complete genome	1437	1437	100%	0.0	99%	CP016586.1
Salmonella enterica subsp. enterica serovar Heidelberg strain SH14-009, complete genome	1437	1437	100%	0.0	99%	CP016581.1
Salmonella enterica subsp. enterica serovar Heidelberg strain SH13-006, complete genome	1437	1437	100%	0.0	99%	CP016579.1
Salmonella enterica subsp. enterica serovar						

Heidelberg strain AMR588-04-00437, complete genome	1437	1437	100%	0.0	99%	CP016576.1
Salmonella enterica subsp. enterica serovar Heidelberg strain AMR588-04-00435, complete genome	1437	1437	100%	0.0	99%	CP016573.1
Salmonella enterica subsp. enterica serovar Heidelberg strain AMR588-04-00320, complete genome	1437	1437	100%	0.0	99%	CP016569.1
Salmonella enterica subsp. enterica serovar Heidelberg strain AMR588-04-00318, complete sequence	1437	1437	100%	0.0	99%	CP016565.1
Salmonella enterica subsp. enterica serovar Heidelberg strain A3EZ223, complete genome	1437	1437	100%	0.0	99%	CP016563.1
Salmonella enterica subsp. enterica serovar Heidelberg strain A3ES40, complete genome	1437	1437	100%	0.0	99%	CP016561.1
Salmonella enterica subsp. enterica serovar Heidelberg strain 09-036813-1A, complete genome	1437	1437	100%	0.0	99%	CP016525.1
Salmonella enterica subsp. enterica serovar Heidelberg strain SA01AB09084001, complete genome	1437	1437	100%	0.0	99%	CP016530.1
Salmonella enterica subsp. enterica serovar Heidelberg strain SA02DT09004001, complete genome	1437	1437	100%	0.0	99%	CP016521.1
Salmonella enterica subsp. enterica serovar Heidelberg strain CE-R2-11-0435, complete genome	1437	1437	100%	0.0	99%	CP016517.1
Salmonella enterica subsp. enterica serovar Heidelberg strain 11-004736-1-7, complete genome	1437	1437	100%	0.0	99%	CP016514.1
Salmonella enterica subsp. enterica serovar Heidelberg strain SH14-028, complete genome	1437	1437	100%	0.0	99%	CP016510.1
Salmonella enterica subsp. enterica serovar Heidelberg strain SH12-003, complete genome	1437	1437	100%	0.0	99%	CP016507.1
Salmonella enterica subsp. enterica serovar Heidelberg strain SH12-007, complete genome	1437	1437	100%	0.0	99%	CP016504.1
Salmonella enterica subsp. enterica serovar Heidelberg strain N13-01290, complete genome	1437	1437	100%	0.0	99%	CP012930.1
Salmonella enterica subsp. enterica serovar Heidelberg strain 12-4374, complete genome	1437	1437	100%	0.0	99%	CP012924.1
Salmonella enterica subsp. enterica serovar Heidelberg strain SA02DT10168701, complete genome	1437	1437	100%	0.0	99%	CP012921.1
Salmonella enterica subsp. enterica serovar Heidelberg str. CFSAN002064, complete genome	1437	1437	100%	0.0	99%	CP005995.1
Salmonella enterica subsp. enterica serovar Heidelberg str. CFSAN002069, complete genome	1437	1437	100%	0.0	99%	CP005390.2
Salmonella enterica subsp. enterica serovar Heidelberg str. 41578, complete genome	1437	1437	100%	0.0	99%	CP004086.1
Salmonella enterica subsp. enterica serovar Heidelberg str. B182, complete genome	1437	1437	100%	0.0	99%	CP003416.1
Salmonella enterica subsp. enterica serovar Heidelberg str. SL476, complete genome	1437	1437	100%	0.0	99%	CP001120.1
Salmonella enterica subsp. enterica serovar Bardo strain SA20113257, complete genome	1393	1393	100%	0.0	98%	CP019404.1
Salmonella enterica subsp. enterica serovar Newport strain CFSAN001660, complete genome	1393	1393	100%	0.0	98%	CP016010.1

Salmonella enterica subsp. enterica serovar Newport str. Levine 15, complete genome	1393	1393	100%	0.0	98%	CP015924.1
Salmonella enterica subsp. enterica serovar Newport str. Levine 1, complete genome	1393	1393	100%	0.0	98%	CP015923.1
Salmonella enterica subsp. enterica serovar Newport str. USDA-ARS-USMARC-1927, complete genome	1393	1393	100%	0.0	98%	CP007216.1
Salmonella enterica subsp. enterica serovar Bareilly str. CFSAN000189, complete genome	1393	1393	100%	0.0	98%	CP006053.1
Salmonella enterica subsp. enterica serovar Newport strain CFSAN003387, complete genome	1387	1387	100%	0.0	98%	CP016014.1
Salmonella enterica subsp. enterica serovar Newport str. CDC 2010K-2159, complete genome	1382	1382	100%	0.0	98%	CP007559.2
Salmonella enterica subsp. enterica serovar Typhimurium strain FORC_020, complete genome	1380	1380	99%	0.0	98%	CP012144.1
Salmonella enterica strain MFDS1004024 chromosome, complete genome	1376	1376	100%	0.0	98%	CP025745.1
Salmonella enterica subsp. enterica serovar Saintpaul strain SGB23 chromosome, complete genome	1376	1376	100%	0.0	98%	CP023166.1
Salmonella enterica subsp. enterica serovar Hvittingfoss str. SA20014981 chromosome, complete genome	1376	1376	100%	0.0	98%	CP022503.1
Salmonella enterica subsp. enterica serovar Manhattan strain SA20084699 chromosome, complete genome	1376	1376	100%	0.0	98%	CP022497.1
Salmonella enterica subsp. enterica serovar Saintpaul strain SA20031783 chromosome, complete genome	1376	1376	100%	0.0	98%	CP022491.1
Salmonella enterica subsp. enterica serovar Braenderup strain SA20026289 chromosome, complete genome	1376	1376	100%	0.0	98%	CP022490.1
Salmonella enterica subsp. enterica serovar Yovokome str. S-1850, complete genome	1376	1376	100%	0.0	98%	CP019418.1
Salmonella enterica subsp. enterica serovar Saintpaul strain CFSAN004174, complete genome	1376	1376	100%	0.0	98%	CP019206.1
Salmonella enterica subsp. enterica serovar Saintpaul strain CFSAN004173, complete genome	1376	1376	100%	0.0	98%	CP019204.1
Salmonella enterica subsp. enterica serovar Thompson strain CFSAN000738, complete genome	1376	1376	100%	0.0	98%	CP019196.1
Salmonella enterica subsp. enterica serovar Saintpaul strain CFSAN004175, complete genome	1376	1376	100%	0.0	98%	CP019172.1
Salmonella enterica subsp. enterica serovar Stanleyville str. CFSAN000624 strain SARB61, complete genome	1376	1376	100%	0.0	98%	CP017723.1
Salmonella enterica subsp. enterica serovar Thompson strain RM1986, complete genome	1376	1376	100%	0.0	98%	CP012514.1
Salmonella enterica subsp. enterica serovar Thompson strain RM1984, complete genome	1376	1376	100%	0.0	98%	CP012513.1
Salmonella enterica subsp. enterica serovar Choleraesuis str. ATCC 10708, complete genome	1376	1376	100%	0.0	98%	CP012344.1
Salmonella enterica subsp. enterica serovar Thompson str. ATCC 8391, complete genome	1376	1376	100%	0.0	98%	CP011396.1
Salmonella enterica subsp. enterica serovar Thompson str. RM6836, complete genome	1376	1376	100%	0.0	98%	CP006717.1
Salmonella enterica subsp. enterica serovar Paratyphi C strain RKS4594, complete	1376	1376	100%	0.0	98%	CP000857.1

genome						
Salmonella enterica subsp. enterica serovar Typhimurium strain AR_0031 chromosome, complete genome	1371	1371	100%	0.0	98%	CP026700.1
Salmonella enterica subsp. enterica serovar Typhimurium strain BL10 chromosome, complete genome	1371	1371	100%	0.0	98%	CP024619.1
Salmonella enterica subsp. enterica serovar Enteritidis strain 92-0392 chromosome, complete genome	1371	1371	100%	0.0	98%	CP018657.1
Salmonella enterica subsp. enterica serovar Typhimurium isolate VNB151-sc-2315230 genome assembly, chromosome: 1	1371	1371	100%	0.0	98%	LT795114.1
Salmonella enterica subsp. enterica serovar Typhimurium strain WW012 chromosome, complete genome	1371	1371	100%	0.0	98%	CP022168.1
Salmonella enterica subsp. enterica serovar Typhimurium strain TW-Stm6 chromosome, complete genome	1371	1371	100%	0.0	98%	CP019649.1
Salmonella enterica subsp. enterica serovar Typhimurium strain FDAARGOS_321, complete genome	1371	1371	100%	0.0	98%	CP022070.1
Salmonella enterica strain FDAARGOS_312, complete genome	1371	1371	100%	0.0	98%	CP022062.1
Salmonella enterica subsp. enterica strain ST1120, complete genome	1371	1371	100%	0.0	98%	CP021909.1
Salmonella enterica subsp. enterica strain 16A242, complete genome	1371	1371	100%	0.0	98%	CP020922.1
Salmonella enterica subsp. enterica serovar Typhimurium isolate STMU2UK genome assembly, chromosome: 1	1371	1371	100%	0.0	98%	LT855376.1
Salmonella enterica strain FORC_030, complete genome	1371	1371	100%	0.0	98%	CP015598.1
Salmonella enterica subsp. enterica serovar Typhimurium strain RM10607, complete genome	1371	1371	100%	0.0	98%	CP013720.1
Salmonella enterica subsp. enterica serovar Typhimurium str. USDA-ARS-USMARC-1899, complete genome	1371	1371	100%	0.0	98%	CP007235.2
Salmonella enterica subsp. enterica serovar Typhimurium str. USDA-ARS-USMARC-1898, complete genome	1371	1371	100%	0.0	98%	CP014971.2
Salmonella enterica subsp. enterica serovar Typhimurium str. CDC H2662, complete genome	1371	1371	100%	0.0	98%	CP014979.2
Salmonella enterica subsp. enterica serovar Typhimurium str. USDA-ARS-USMARC-1810, complete genome	1371	1371	100%	0.0	98%	CP014982.2
Salmonella enterica subsp. enterica serovar Typhimurium strain 81741, complete genome	1371	1371	100%	0.0	98%	CP019442.1
Salmonella enterica subsp. enterica serovar Dublin str. ATCC 39184, complete genome	1371	1371	100%	0.0	98%	CP019179.1
Salmonella enterica subsp. enterica serovar Typhimurium strain RM10961, complete genome	1371	1371	100%	0.0	98%	CP013702.1
Salmonella enterica subsp. enterica serovar Typhimurium strain 22792, complete genome	1371	1371	100%	0.0	98%	CP017621.1
Salmonella enterica subsp. enterica serovar Typhimurium strain 22495, complete genome	1371	1371	100%	0.0	98%	CP017617.1
Salmonella enterica subsp. enterica serovar Typhimurium str. SARA13, complete genome	1371	1371	100%	0.0	98%	CP017728.1
Salmonella enterica subsp. enterica serovar Typhimurium strain 13-931, complete genome	1371	1371	100%	0.0	98%	CP016385.1

Salmonella enterica subsp. enterica serovar Typhimurium strain NC983, complete genome	1371	1371	100%	0.0	98%	CP015157.1
Salmonella enterica subsp. enterica strain SA972816, complete genome	1371	1371	100%	0.0	98%	CP007484.1
Salmonella enterica subsp. enterica serovar Typhimurium str. USDA-ARS-USMARC-1896, complete genome	1371	1371	100%	0.0	98%	CP014977.1
Salmonella enterica subsp. enterica serovar Typhimurium str. CDC 2009K-1640, complete genome	1371	1371	100%	0.0	98%	CP014975.1
Salmonella enterica subsp. enterica serovar Typhimurium str. USDA-ARS-USMARC-1808, complete genome	1371	1371	100%	0.0	98%	CP014969.1
Salmonella enterica subsp. enterica serovar Typhimurium str. CDC 2011K-1702, complete genome	1371	1371	100%	0.0	98%	CP014967.1
Salmonella enterica subsp. enterica serovar Typhimurium str. CDC 2010K-1587, complete genome	1371	1371	100%	0.0	98%	CP014965.1
Salmonella enterica subsp. enterica serovar Typhimurium str. CDC 2009K-2059, complete genome	1371	1371	100%	0.0	98%	CP014983.1
Salmonella enterica subsp. enterica serovar Typhimurium str. USDA-ARS-USMARC-1880, complete genome	1371	1371	100%	0.0	98%	CP014981.1
Salmonella enterica subsp. enterica serovar Typhimurium strain RM9437, complete genome	1371	1371	100%	0.0	98%	CP012985.1
Salmonella enterica subsp. enterica serovar Typhimurium strain SL1344RX genome	1371	1371	100%	0.0	98%	CP011233.1
Salmonella enterica subsp. enterica serovar Typhimurium strain YU15, complete genome	1371	1371	100%	0.0	98%	CP014358.1
Salmonella enterica subsp. enterica serovar Typhimurium strain SO2, complete genome	1371	1371	100%	0.0	98%	CP014356.1
Salmonella enterica subsp. enterica serovar Typhimurium strain SO3, complete genome	1371	1371	100%	0.0	98%	CP014536.1
Salmonella enterica strain LT2, complete genome	1371	1371	100%	0.0	98%	CP014051.1
Salmonella enterica subsp. enterica serovar Typhimurium isolate SO4698-09 genome assembly, chromosome: I	1371	1371	100%	0.0	98%	LN999997.1
Salmonella enterica subsp. enterica serovar Typhimurium str. LT2, complete genome	1371	1371	100%	0.0	98%	AE006468.2
Salmonella enterica subsp. enterica serovar Typhimurium strain 33676, complete genome	1371	1371	100%	0.0	98%	CP012681.1
Salmonella enterica subsp. enterica strain YU39, complete genome	1371	1371	100%	0.0	98%	CP011428.1
Salmonella enterica subsp. enterica serovar Typhimurium str. CDC 2011K-0870, complete genome	1371	1371	100%	0.0	98%	CP007523.1
Salmonella enterica subsp. enterica serovar Typhimurium strain ATCC 13311, complete genome	1371	1371	100%	0.0	98%	CP009102.1
Salmonella enterica subsp. enterica serovar Choleraesuis strain C500, complete genome	1371	1371	100%	0.0	98%	CP007639.1
Salmonella enterica subsp. enterica serovar Typhimurium strain VNP20009, complete genome	1371	1371	100%	0.0	98%	CP007804.2
Salmonella enterica subsp. enterica serovar Dublin genome assembly SC50_1, chromosome : I	1371	1371	100%	0.0	98%	LK931502.1
Salmonella enterica subsp. enterica serovar Typhimurium str. L-3553 DNA, complete genome	1371	1371	100%	0.0	98%	AP014565.1
Salmonella enterica subsp. enterica serovar						

Typhimurium strain 138736, complete genome	1371	1371	100%	0.0	98%	CP007581.1
Salmonella enterica subsp. enterica serovar Bovismorbificans str. 3114 complete genome	1371	1371	100%	0.0	98%	HF969015.2
Salmonella enterica subsp. enterica serovar Typhimurium str. DT2, complete genome	1371	1371	100%	0.0	98%	HG326213.1
Salmonella enterica subsp. enterica serovar Typhimurium DT104 main chromosome, complete genome	1371	1371	100%	0.0	98%	HF937208.1
Salmonella enterica subsp. enterica serovar Typhimurium str. 08-1736, complete genome	1371	1371	100%	0.0	98%	CP006602.1
Salmonella enterica subsp. enterica serovar Typhimurium var. 5- str. CFSAN001921, complete genome	1371	1371	100%	0.0	98%	CP006048.1
Salmonella enterica subsp. enterica serovar Typhimurium str. U288, complete genome	1371	1371	100%	0.0	98%	CP003836.1
Salmonella enterica subsp. enterica serovar Typhimurium str. 798, complete genome	1371	1371	100%	0.0	98%	CP003386.1
Salmonella enterica subsp. enterica serovar Typhimurium str. UK-1, complete genome	1371	1371	100%	0.0	98%	CP002614.1
Salmonella enterica subsp. enterica serovar Typhimurium str. ST4/74, complete genome	1371	1371	100%	0.0	98%	CP002487.1
Salmonella enterica subsp. enterica serovar Typhimurium str. T000240 DNA, complete genome	1371	1371	100%	0.0	98%	AP011957.1
Salmonella enterica subsp. enterica serovar Typhimurium SL1344 complete genome	1371	1371	100%	0.0	98%	FQ312003.1
Salmonella enterica subsp. enterica serovar Typhimurium str. 14028S, complete genome	1371	1371	100%	0.0	98%	CP001363.1
Salmonella enterica subsp. enterica serovar Typhimurium str. D23580 complete genome	1371	1371	100%	0.0	98%	FN424405.1
Salmonella enterica subsp. enterica serovar Dublin str. CT_02021853, complete genome	1371	1371	100%	0.0	98%	CP001144.1
Salmonella enterica subsp. enterica serovar Paratyphi B str. SPB7, complete genome	1371	1371	100%	0.0	98%	CP000886.1
Salmonella typhimurium exonuclease VII (xseA), ShdA (shdA), RatC (ratC), RatB (ratB), RatA (ratA), SinI (sinI), and SinH (sinH) genes, complete cds; and YfgK (yfgK) gene, partial cds	1371	1371	100%	0.0	98%	AF140550.2
Salmonella enterica subsp. enterica serovar Choleraesuis str. SC-B67, complete genome	1371	1371	100%	0.0	98%	AE017220.1
Salmonella enterica subsp. enterica serovar Saintpaul strain FDAARGOS_373 chromosome, complete genome	1365	1365	100%	0.0	98%	CP023512.1
Salmonella enterica subsp. enterica serovar Java strain NCTC5706 genome assembly, chromosome: 1	1365	1365	100%	0.0	98%	LT571437.1
Salmonella enterica subsp. enterica strain 08-00436, complete genome	1256	1256	99%	0.0	95%	CP020492.1
Salmonella enterica subsp. enterica serovar Senftenberg strain N17-509 chromosome, complete genome	1249	1249	100%	0.0	95%	CP026379.1
Salmonella enterica subsp. enterica serovar Mbandaka strain SA20026234 chromosome, complete genome	1249	1249	100%	0.0	95%	CP022489.1
Salmonella enterica subsp. enterica serovar Senftenberg strain 775W, complete genome	1249	1249	100%	0.0	95%	CP016837.1
Salmonella enterica subsp. enterica serovar Senftenberg str. ATCC 43845, complete genome	1249	1249	100%	0.0	95%	CP019194.1
Salmonella enterica subsp. enterica serovar Typhimurium strain FORC_015, complete genome	1249	1249	100%	0.0	95%	CP011365.1

Salmonella enterica subsp. enterica serovar Ouakam str. SA20034636, complete genome	1236	1236	99%	0.0	95%	CP022116.1
Salmonella enterica subsp. enterica serovar Inverness str. ATCC 10720, complete genome	1236	1236	99%	0.0	95%	CP019181.1
Salmonella enterica subsp. enterica serovar Ouakam strain GNT-01 genome	1236	1236	99%	0.0	95%	CP012038.1
Salmonella enterica subsp. enterica serovar Hayindogo strain CFSAN050752 chromosome, complete genome	1230	1230	99%	0.0	95%	CP017719.1
Salmonella enterica subsp. enterica serovar Anatum str. USDA-ARS-USMARC-1781, complete genome	1230	1230	99%	0.0	95%	CP014666.2
Salmonella enterica subsp. enterica serovar Anatum str. CDC 06-0532, complete genome	1230	1230	99%	0.0	95%	CP007211.2
Salmonella enterica subsp. enterica serovar Anatum str. USDA-ARS-USMARC-1735, complete genome	1230	1230	99%	0.0	95%	CP007584.2
Salmonella enterica subsp. enterica serovar Anatum str. ATCC BAA-1592, complete genome	1230	1230	99%	0.0	95%	CP007531.1
Salmonella enterica subsp. enterica serovar Krefeld str. SA20030536, complete genome	1229	1229	99%	0.0	95%	CP019413.1
Salmonella enterica subsp. enterica serovar Anatum str. USDA-ARS-USMARC-1765, complete genome	1225	1225	99%	0.0	95%	CP014659.2
Salmonella enterica subsp. enterica serovar Anatum str. USDA-ARS-USMARC-1677, complete genome	1225	1225	99%	0.0	95%	CP014663.2
Salmonella enterica subsp. enterica serovar Anatum str. USDA-ARS-USMARC-1175, complete genome	1225	1225	99%	0.0	95%	CP007483.2
Salmonella enterica subsp. enterica serovar Anatum str. USDA-ARS-USMARC-1727, complete genome	1225	1225	99%	0.0	95%	CP014621.2
Salmonella enterica subsp. enterica serovar Borreze str. SA20041063, complete genome	1225	1225	99%	0.0	95%	CP019407.1
Salmonella enterica subsp. enterica serovar Anatum str. USDA-ARS-USMARC-1766, complete genome	1225	1225	99%	0.0	95%	CP014665.1
Salmonella enterica subsp. enterica serovar Anatum str. USDA-ARS-USMARC-1728, complete genome	1225	1225	99%	0.0	95%	CP014664.1
Salmonella enterica subsp. enterica serovar Anatum str. USDA-ARS-USMARC-1783, complete sequence	1225	1225	99%	0.0	95%	CP014661.1
Salmonella enterica subsp. enterica serovar Anatum str. USDA-ARS-USMARC-1736, complete genome	1225	1225	99%	0.0	95%	CP014657.1
Salmonella enterica subsp. enterica serovar Anatum str. USDA-ARS-USMARC-1676, complete genome	1225	1225	99%	0.0	95%	CP014620.1
Salmonella enterica subsp. enterica serovar Agona str. 392869-2, complete genome	1225	1225	99%	0.0	95%	CP015024.1
Salmonella enterica subsp. enterica serovar Anatum strain GT-38, complete genome	1225	1225	99%	0.0	95%	CP013226.1
Salmonella enterica subsp. enterica serovar Anatum strain GT-01, complete genome	1225	1225	99%	0.0	95%	CP013222.1
Salmonella enterica subsp. enterica serovar Agona str. 460004 2-1, complete genome	1225	1225	99%	0.0	95%	CP011259.1
Salmonella enterica subsp. enterica serovar Agona str. 24249, complete genome	1225	1225	99%	0.0	95%	CP006876.1
Salmonella enterica subsp. enterica serovar Agona str. SL483, complete genome	1225	1225	99%	0.0	95%	CP001138.1
Salmonella enterica subsp. enterica serovar Senftenberg genome assembly NCTC10384,	1221	1221	100%	0.0	94%	LN868943.1

chromosome : 1						
Salmonella enterica strain FDAARGOS_313, complete genome	1219	1219	99%	0.0	95%	CP022069.1
Salmonella enterica subsp. enterica serovar Kentucky str. SA20030505 chromosome, complete genome	1210	1210	100%	0.0	94%	CP022500.1
Salmonella enterica subsp. enterica serovar Derby isolate 2014LSAL01779 chromosome, complete genome	1205	1205	100%	0.0	94%	CP026609.1
Salmonella enterica strain FORC_038, complete genome	1205	1205	100%	0.0	94%	CP015574.1
Salmonella enterica subsp. enterica serovar Apapa str. SA20060561, complete genome	1203	1203	99%	0.0	94%	CP019403.1
Salmonella enterica subsp. enterica serovar Kentucky strain PU131 chromosome, complete genome	1199	1199	100%	0.0	94%	CP026327.1
Salmonella enterica subsp. enterica serovar Infantis strain CFSAN003307 chromosome, complete genome	1199	1199	100%	0.0	94%	CP019202.1
Salmonella enterica subsp. enterica serovar Infantis strain N55391, complete genome	1199	1199	100%	0.0	94%	CP016410.1
Salmonella enterica subsp. enterica serovar Infantis strain FSIS1502916, complete genome	1199	1199	100%	0.0	94%	CP016408.1
Salmonella enterica subsp. enterica serovar Infantis strain FSIS1502169, complete genome	1199	1199	100%	0.0	94%	CP016406.1
Salmonella enterica subsp. enterica serovar Infantis strain CVM44454, complete genome	1199	1199	100%	0.0	94%	CP016412.1
Salmonella enterica subsp. enterica serovar Infantis genome assembly SINFA, chromosome : 1	1199	1199	100%	0.0	94%	LN649235.1
Salmonella enterica strain CFSAN024439 chromosome, complete genome	1197	1197	99%	0.0	94%	CP024169.1
Salmonella enterica subsp. enterica serovar Tennessee strain CFSAN070643 chromosome, complete genome	1197	1197	99%	0.0	94%	CP024168.1
Salmonella enterica subsp. enterica serovar Tennessee strain CFSAN070645 chromosome, complete genome	1197	1197	99%	0.0	94%	CP024164.1
Salmonella enterica subsp. enterica serovar Albany str. ATCC 51960, complete genome	1197	1197	99%	0.0	94%	CP019177.1
Salmonella enterica subsp. enterica serovar Tennessee strain CFSAN001387, complete genome	1197	1197	99%	0.0	94%	CP014994.1
Salmonella enterica subsp. enterica serovar Tennessee str. TXSC_TXSC08-19, complete genome	1197	1197	99%	0.0	94%	CP007505.1
Salmonella enterica subsp. enterica Serovar Cubana str. CFSAN002050, complete genome	1197	1197	99%	0.0	94%	CP006055.1
Salmonella enterica subsp. enterica serovar Bergen str. ST350, complete genome	1194	1194	100%	0.0	94%	CP019405.1
Salmonella enterica subsp. enterica serovar Derby strain SA20035215 chromosome, complete genome	1186	1186	99%	0.0	94%	CP022494.1
Salmonella enterica subsp. enterica serovar Hillingdon str. N1529-D3, complete genome	1131	1131	95%	0.0	94%	CP019410.1
Salmonella enterica subsp. enterica serovar Weltevreden str. 1655, complete genome	1092	1092	97%	0.0	92%	CP014996.1
Salmonella enterica subsp. enterica serovar Weltevreden genome assembly 99_3134, chromosome : 1	1092	1092	97%	0.0	92%	LN890524.1
Salmonella enterica subsp. enterica serovar Weltevreden genome assembly 98_11262, chromosome : 1	1092	1092	97%	0.0	92%	LN890522.1
Salmonella enterica subsp. enterica serovar Weltevreden genome assembly C2346,	1092	1092	97%	0.0	92%	LN890520.1

chromosome : 1						
Salmonella enterica subsp. enterica serovar Weltevreden genome assembly 10259, chromosome : 1	1092	1092	97%	0.0	92%	LN890518.1
Salmonella enterica subsp. enterica serovar Weltevreden str. 2007-60-3289-1 complete genome, contig 43	1092	1092	97%	0.0	92%	FR775230.1
Salmonella enterica subsp. enterica serovar Paratyphi A strain FDAARGOS_368 chromosome, complete genome	1031	1031	97%	0.0	91%	CP023508.1
Salmonella enterica subsp. enterica serovar Paratyphi A str. ATCC 11511, complete genome	1031	1031	97%	0.0	91%	CP019185.1
Salmonella enterica subsp. enterica serovar Paratyphi A strain CMCC50093 genome	1031	1031	97%	0.0	91%	CP011967.1
Salmonella enterica subsp. enterica serovar Paratyphi A strain CMCC 50973, complete genome	1031	1031	97%	0.0	91%	CP009049.1
Salmonella enterica subsp. enterica serovar Paratyphi A strain CMCC 50503, complete genome	1031	1031	97%	0.0	91%	CP009559.1
Salmonella enterica subsp. enterica serovar Paratyphi A str. AKU_12601 complete genome, strain AKU_12601	1031	1031	97%	0.0	91%	FM200053.1
Salmonella enterica subsp. enterica serovar Paratyphi A str. ATCC 9150, complete genome	1031	1031	97%	0.0	91%	CP000026.1
Salmonella enterica subsp. enterica serovar Saintpaul str. SARA26, complete genome	1009	1009	97%	0.0	90%	CP017727.1
Salmonella enterica subsp. enterica serovar Mbandaka str. ATCC 51958, complete genome	992	992	99%	0.0	89%	CP019183.1
Salmonella enterica subsp. enterica serovar Typhi strain SGB90 genome assembly, chromosome: 1	987	987	97%	0.0	90%	LT904870.2
Salmonella enterica subsp. enterica serovar Typhi strain SGB89 genome assembly, chromosome: 1	987	987	97%	0.0	90%	LT904882.1
Salmonella enterica subsp. enterica serovar Typhi strain OVG_041 genome assembly, chromosome: 2	981	981	97%	0.0	90%	LT906560.1
Salmonella enterica subsp. enterica serovar Typhi isolate ISP_03_07467_SGB110-sc-1979083 genome assembly, chromosome: 1	981	981	97%	0.0	90%	LT905060.2
Salmonella enterica subsp. enterica serovar Typhi strain 1036491 genome assembly, chromosome: 1	981	981	97%	0.0	90%	LT906495.1
Salmonella enterica subsp. enterica serovar Typhi strain ERL024120 genome assembly, chromosome: 1	981	981	97%	0.0	90%	LT906494.1
Salmonella enterica subsp. enterica serovar Typhi isolate 1554-sc-2165329 genome assembly, chromosome: 1	981	981	97%	0.0	90%	LT906493.1
Salmonella enterica subsp. enterica serovar Typhi isolate E98_3139-sc-1927833 genome assembly, chromosome: 1	981	981	97%	0.0	90%	LT905143.1
Salmonella enterica subsp. enterica serovar Typhi strain H12ESR00755-001A genome assembly, chromosome: 1	981	981	97%	0.0	90%	LT905142.1
Salmonella enterica subsp. enterica serovar Typhi strain OVG_041 genome assembly, chromosome: 1	981	981	97%	0.0	90%	LT905141.1
Salmonella enterica subsp. enterica serovar Typhi strain ERL082356 genome assembly, chromosome: 1	981	981	97%	0.0	90%	LT905140.1
Salmonella enterica subsp. enterica serovar Typhi strain 2010-007898 genome assembly, chromosome: 1	981	981	97%	0.0	90%	LT905139.1

Salmonella enterica subsp. enterica serovar Typhi strain ty3-243 genome assembly, chromosome: 2	981	981	97%	0.0	90%	LT905090.1
Salmonella enterica subsp. enterica serovar Typhi strain ERL024120 genome assembly, chromosome: 1	981	981	97%	0.0	90%	LT905088.1
Salmonella enterica subsp. enterica serovar Typhi isolate 1554-sc-2165329 genome assembly, chromosome: 1	981	981	97%	0.0	90%	LT905064.1
Salmonella enterica subsp. enterica serovar Typhi isolate lupe_GEN0059-sc-1979081 genome assembly, chromosome: 1	981	981	97%	0.0	90%	LT905063.1
Salmonella enterica subsp. enterica serovar Typhi isolate 403Ty-sc-1979084 genome assembly, chromosome: 1	981	981	97%	0.0	90%	LT905062.1
Salmonella enterica subsp. enterica serovar Typhi strain ERL12960 genome assembly, chromosome: 1	981	981	97%	0.0	90%	LT904894.1
Salmonella enterica subsp. enterica serovar Typhi strain 1016889 genome assembly, chromosome: 1	981	981	97%	0.0	90%	LT904893.1
Salmonella enterica subsp. enterica serovar Typhi strain 80-2002 genome assembly, chromosome: 1	981	981	97%	0.0	90%	LT904891.1
Salmonella enterica subsp. enterica serovar Typhi strain H12ESR00394-001A genome assembly, chromosome: 1	981	981	97%	0.0	90%	LT904890.1
Salmonella enterica subsp. enterica serovar Typhi strain 129-0238-M genome assembly, chromosome: 1	981	981	97%	0.0	90%	LT904888.1
Salmonella enterica subsp. enterica serovar Typhi strain 76-1292 genome assembly, chromosome: 1	981	981	97%	0.0	90%	LT904887.1
Salmonella enterica subsp. enterica serovar Typhi strain SGB82 genome assembly, chromosome: 1	981	981	97%	0.0	90%	LT904886.1
Salmonella enterica subsp. enterica serovar Typhi strain 1036491 genome assembly, chromosome: 1	981	981	97%	0.0	90%	LT904885.1
Salmonella enterica subsp. enterica serovar Typhi strain ERL041834 genome assembly, chromosome: 1	981	981	97%	0.0	90%	LT904884.1
Salmonella enterica subsp. enterica serovar Typhi strain ERL103914 genome assembly, chromosome: 1	981	981	97%	0.0	90%	LT904883.1
Salmonella enterica subsp. enterica serovar Typhi strain UI2120 genome assembly, chromosome: 1	981	981	97%	0.0	90%	LT904881.1
Salmonella enterica subsp. enterica serovar Typhi strain ty3-193 genome assembly, chromosome: 1	981	981	97%	0.0	90%	LT904878.1
Salmonella enterica subsp. enterica serovar Typhi strain SGB92 genome assembly, chromosome: 1	981	981	97%	0.0	90%	LT904877.1
Salmonella enterica subsp. enterica serovar Typhi strain ERL114000 genome assembly, chromosome: 1	981	981	97%	0.0	90%	LT904876.1
Salmonella enterica subsp. enterica serovar Typhi strain ERL024919 genome assembly, chromosome: 1	981	981	97%	0.0	90%	LT904875.1
Salmonella enterica subsp. enterica serovar Typhi strain ERL11909 genome assembly, chromosome: 1	981	981	97%	0.0	90%	LT904872.1
Salmonella enterica subsp. enterica serovar Typhi strain 1553 genome assembly, chromosome: 1	981	981	97%	0.0	90%	LT904871.1
Salmonella enterica subsp. enterica serovar Typhi strain ERL052042 genome assembly, chromosome: 1	981	981	97%	0.0	90%	LT904869.1
Salmonella enterica subsp. enterica serovar						

Typhi strain H12ESR04734-001A genome assembly, chromosome: 1	981	981	97%	0.0	90%	LT904868.1
Salmonella enterica subsp. enterica serovar Typhi strain ERL034151 genome assembly, chromosome: 1	981	981	97%	0.0	90%	LT904867.1
Salmonella enterica subsp. enterica serovar Typhi strain M223 genome assembly, chromosome: 1	981	981	97%	0.0	90%	LT904854.1
Salmonella enterica subsp. enterica serovar Typhi strain TY585 genome assembly, chromosome: 1	981	981	97%	0.0	90%	LT904852.1
Salmonella enterica subsp. enterica serovar Typhi strain ERL072973 genome assembly, chromosome: 1	981	981	97%	0.0	90%	LT904777.1
Salmonella enterica subsp. enterica serovar Waycross strain SA20041608, complete genome	981	981	97%	0.0	89%	CP022138.1
Salmonella enterica subsp. enterica serovar Typhi strain ERL12148 genome assembly, chromosome: 1	981	981	97%	0.0	90%	LT883153.1
Salmonella enterica subsp. enterica serovar Typhi strain BL60006 genome assembly, chromosome: 1	981	981	97%	0.0	90%	LT882486.1
Salmonella enterica subsp. enterica serovar Typhi strain B/SF/13/03/195, complete genome	981	981	97%	0.0	90%	CP012151.1
Salmonella enterica subsp. enterica serovar Typhi strain PM016/13, complete genome	981	981	97%	0.0	90%	CP012091.1
Salmonella enterica subsp. enterica serovar Typhi str. CT18, complete chromosome	981	981	97%	0.0	90%	AL513382.1
Salmonella enterica subsp. enterica serovar Typhi str. Ty21a, complete genome	981	981	97%	0.0	90%	CP002099.1
Salmonella enterica subsp. enterica serovar Typhi str. P-stx-12, complete genome	981	981	97%	0.0	90%	CP003278.1
Salmonella enterica subsp. enterica serovar Typhi Ty2, complete genome	981	981	97%	0.0	90%	AE014613.1
Salmonella enterica subsp. enterica serovar Cerro strain 87, complete genome	970	970	97%	0.0	89%	CP008925.1
Salmonella enterica subsp. enterica serovar Cerro str. CFSAN001588, complete genome	970	970	97%	0.0	89%	CP012833.1
Salmonella enterica subsp. enterica serovar Djakarta str. S-1087, complete genome	966	966	97%	0.0	89%	CP019409.1
Salmonella enterica subsp. enterica serovar Manchester str. ST278, complete genome	948	948	97%	0.0	89%	CP019414.1
Salmonella enterica subsp. enterica serovar Muenster str. 420 strain CFSAN001301 chromosome, complete genome	861	861	97%	0.0	87%	CP019201.1
Salmonella enterica subsp. enterica serovar Muenster str. 0315 strain 315, complete genome	861	861	97%	0.0	87%	CP019198.1
Salmonella enterica subsp. enterica serovar Minnesota strain CFSAN017963, complete genome	856	856	97%	0.0	87%	CP017720.1
Salmonella enterica subsp. enterica serovar Montevideo str. USDA-ARS-USMARC-1921, complete genome	856	856	97%	0.0	87%	CP007540.2
Salmonella enterica subsp. enterica serovar Chester str. ATCC 11997, complete genome	856	856	97%	0.0	87%	CP019178.1
Salmonella enterica subsp. enterica serovar Montevideo str. USDA-ARS-USMARC-1903, complete genome	856	856	97%	0.0	87%	CP007222.1
Salmonella enterica subsp. enterica serovar Bredeney str. CFSAN001080, complete genome	856	856	97%	0.0	87%	CP007533.1
Salmonella enterica subsp. enterica serovar Montevideo str. 507440-20, complete genome	856	856	97%	0.0	87%	CP007530.1

Salmonella enterica subsp. enterica serovar Schwarzengrund str. CVM19633, complete genome	856	856	97%	0.0	87%	CP001127.1
Salmonella enterica subsp. enterica serovar Poona str. ATCC BAA-1673 chromosome, complete genome	850	850	97%	0.0	87%	CP019189.1
Salmonella enterica subsp. enterica serovar Pomona str. ATCC 10729 chromosome, complete genome	850	850	97%	0.0	87%	CP019186.1
Salmonella enterica subsp. enterica serovar Give strain CFSAN024229 chromosome, complete genome	850	850	97%	0.0	87%	CP019174.1
Salmonella enterica subsp. enterica strain 2012K-0678, genome	850	850	97%	0.0	87%	CP020718.1
Salmonella enterica subsp. enterica serovar Minnesota str. ATCC 49284, complete genome	850	850	97%	0.0	87%	CP019184.1
Salmonella enterica subsp. enterica serovar Oranienburg str. 0250 strain CFSAN001285 chromosome, complete genome	845	845	97%	0.0	86%	CP019197.1
Salmonella enterica subsp. enterica serovar Gaminara strain CFSAN070644 chromosome, complete genome	845	845	97%	0.0	86%	CP024165.1
Salmonella enterica subsp. enterica serovar Onderstepoort str. SA20060086, complete genome	845	845	97%	0.0	86%	CP022034.1
Salmonella enterica subsp. enterica serovar Johannesburg str. ST203, complete genome	845	845	97%	0.0	86%	CP019411.1
Salmonella enterica subsp. enterica strain RM11065 chromosome, complete genome	839	839	97%	0.0	86%	CP022663.1
Salmonella enterica subsp. enterica serovar Quebec str. S-1267, complete genome	839	839	97%	0.0	86%	CP022019.1
Salmonella enterica subsp. enterica serovar Antsalova str. S01-0511, complete genome	839	839	97%	0.0	86%	CP019116.1
Salmonella enterica subsp. enterica serovar Abaetetuba str. ATCC 35640, complete genome	839	839	97%	0.0	86%	CP007532.1
Salmonella enterica subsp. enterica serovar India str. SA20085604, complete genome	833	833	97%	0.0	86%	CP022015.1
Salmonella enterica subsp. enterica serovar Koessen str. S-1501, complete genome	833	833	97%	0.0	86%	CP019412.1
Salmonella enterica subsp. enterica serovar Panama str. ATCC 7378, complete genome	833	833	97%	0.0	86%	CP012346.1
Salmonella enterica subsp. enterica serovar Rubislaw str. ATCC 10717, complete genome	828	828	97%	0.0	86%	CP019192.1
Salmonella enterica subsp. enterica serovar Javiana str. CFSAN001992, complete genome	822	822	97%	0.0	86%	CP004027.1
Salmonella enterica subsp. enterica serovar Sloterdijk str. ATCC 15791, complete genome	795	795	97%	0.0	85%	CP012349.1
Salmonella enterica subsp. enterica serovar Indiana strain D90, complete genome	688	688	66%	0.0	90%	CP022450.1
Salmonella enterica strain C629, complete genome	688	688	66%	0.0	90%	CP015724.1

Alignments

Salmonella enterica strain MFDS1004839 chromosome, complete genome
Sequence ID: **CP026569.1** Length: 4679649 Number of Matches: 1
Range 1: 1273425 to 1274214

Score	Expect	Identities	Gaps	Strand	Frame
1459 bits(790)	0.0()	790/790(100%)	0/790(0%)	Plus/Minus	

Features:

```
Query  1        AATGCTGTGAATCTTGCCGCCGCTGTTGGCGTTATACAGCGCCTCAAGCTGGTCGATGCG  60
                ||||||||||||||||||||||||||||||||||||||||||||||||||||||||||||
Sbjct  1274214  AATGCTGTGAATCTTGCCGCCGCTGTTGGCGTTATACAGCGCCTCAAGCTGGTCGATGCG  1274155

Query  61       CGGCAGGCGGTTCGCCGCGCAGCCGCCCGCATCCGGGTTGCCCACCGCGTCGCTATGGGC  120
                ||||||||||||||||||||||||||||||||||||||||||||||||||||||||||||
Sbjct  1274154  CGGCAGGCGGTTCGCCGCGCAGCCGCCCGCATCCGGGTTGCCCACCGCGTCGCTATGGGC  1274095

Query  121      CACGCGCGCCCAGGTCTCATTGCTCTCGTCCTGGGTACGCAGCGGGTTGCTCACTTCACC  180
                ||||||||||||||||||||||||||||||||||||||||||||||||||||||||||||
Sbjct  1274094  CACGCGCGCCCAGGTCTCATTGCTCTCGTCCTGGGTACGCAGCGGGTTGCTCACTTCACC  1274035

Query  181      CGCCAGTTTGGGCCGCTCAAAAGTCCAGTCGCCCACGGTAATCGTGTCGGCCATATGGCC  240
                ||||||||||||||||||||||||||||||||||||||||||||||||||||||||||||
Sbjct  1274034  CGCCAGTTTGGGCCGCTCAAAAGTCCAGTCGCCCACGGTAATCGTGTCGGCCATATGGCC  1273975

Query  241      CCACATTTTGGCTTTTGCCGTATCCGGGCTGGTCGCGACGGTAAACTTCACGCTGCGGGA  300
                ||||||||||||||||||||||||||||||||||||||||||||||||||||||||||||
Sbjct  1273974  CCACATTTTGGCTTTTGCCGTATCCGGGCTGGTCGCGACGGTAAACTTCACGCTGCGGGA  1273915

Query  301      AACCGGCGTATTGATGTATGAATTGACGGGCACGATGCTTAACGGCGTCAGCAGCCCCAC  360
                ||||||||||||||||||||||||||||||||||||||||||||||||||||||||||||
Sbjct  1273914  AACCGGCGTATTGATGTATGAATTGACGGGCACGATGCTTAACGGCGTCAGCAGCCCCAC  1273855

Query  361      GCCCTTTTTCTGGGTGAGGATAATGGTCGCATCGCCGTTAGCGTCGGTGATCCCGTGATA  420
                ||||||||||||||||||||||||||||||||||||||||||||||||||||||||||||
Sbjct  1273854  GCCCTTTTTCTGGGTGAGGATAATGGTCGCATCGCCGTTAGCGTCGGTGATCCCGTGATA  1273795

Query  421      AACCTGCCCGGCAACGTAGCCCACGTCGTCAAACTGCATCTCGCCGTTGCTGGTATCGGT  480
                ||||||||||||||||||||||||||||||||||||||||||||||||||||||||||||
Sbjct  1273794  AACCTGCCCGGCAACGTAGCCCACGTCGTCAAACTGCATCTCGCCGTTGCTGGTATCGGT  1273735

Query  481      AAAGCCGGTCGTCAGCCCGTCACGCCGCCTGCCGTTCGCCATGGTGATGGTGAAGTCGGT  540
                ||||||||||||||||||||||||||||||||||||||||||||||||||||||||||||
Sbjct  1273734  AAAGCCGGTCGTCAGCCCGTCACGCCGCCTGCCGTTCGCCATGGTGATGGTGAAGTCGGT  1273675

Query  541      ATTGCCGATCGCTATGCCGTTAAGGGCGTTGCGCGAATGGATGTTCATCTTAACCTGTTC  600
                ||||||||||||||||||||||||||||||||||||||||||||||||||||||||||||
Sbjct  1273674  ATTGCCGATCGCTATGCCGTTAAGGGCGTTGCGCGAATGGATGTTCATCTTAACCTGTTC  1273615

Query  601      ACCCACTTTGGCGTCGGCGTTTTTGATCACCGGGTCGTCGTTGAAGTAGACGTCCAGCAC  660
                ||||||||||||||||||||||||||||||||||||||||||||||||||||||||||||
Sbjct  1273614  ACCCACTTTGGCGTCGGCGTTTTTGATCACCGGGTCGTCGTTGAAGTAGACGTCCAGCAC  1273555

Query  661      CGCCACCATTTCATTGGCCGAGCAGGTCAGGTAGTTGGGCGTATCGGTAAATTTGCCGCT  720
                ||||||||||||||||||||||||||||||||||||||||||||||||||||||||||||
Sbjct  1273554  CGCCACCATTTCATTGGCCGAGCAGGTCAGGTAGTTGGGCGTATCGGTAAATTTGCCGCT  1273495

Query  721      ATCGCCGTTTTCGAGGTTGACGTGTACCGTCCCGGAGGCGTCGCTGTCGGCGGCGATGAA  780
                ||||||||||||||||||||||||||||||||||||||||||||||||||||||||||||
Sbjct  1273494  ATCGCCGTTTTCGAGGTTGACGTGTACCGTCCCGGAGGCGTCGCTGTCGGCGGCGATGAA  1273435

Query  781      ATAATCTTCA  790
                ||||||||||
Sbjct  1273434  ATAATCTTCA  1273425
```

Salmonella enterica strain FDAARGOS_70 chromosome, complete genome
Sequence ID: **CP026052.1** Length: 4679420 Number of Matches: 1
Range 1: 370146 to 370935

Score	Expect	Identities	Gaps	Strand	Frame
1459 bits(790)	0.0()	790/790(100%)	0/790(0%)	Plus/Minus	

Features:

```
Query  1       AATGCTGTGAATCTTGCCGCCGCTGTTGGCGTTATACAGCGCCTCAAGCTGGTCGATGCG  60
               ||||||||||||||||||||||||||||||||||||||||||||||||||||||||||||
Sbjct  370935  AATGCTGTGAATCTTGCCGCCGCTGTTGGCGTTATACAGCGCCTCAAGCTGGTCGATGCG  370876

Query  61      CGGCAGGCGGTTCGCCGCGCAGCCGCCCGCATCCGGGTTGCCCACCGCGTCGCTATGGGC  120
               ||||||||||||||||||||||||||||||||||||||||||||||||||||||||||||
Sbjct  370875  CGGCAGGCGGTTCGCCGCGCAGCCGCCCGCATCCGGGTTGCCCACCGCGTCGCTATGGGC  370816

Query  121     CACGCGCGCCCAGGTCTCATTGCTCTCGTCCTGGGTACGCAGCGGGTTGCTCACTTCACC  180
               ||||||||||||||||||||||||||||||||||||||||||||||||||||||||||||
Sbjct  370815  CACGCGCGCCCAGGTCTCATTGCTCTCGTCCTGGGTACGCAGCGGGTTGCTCACTTCACC  370756

Query  181     CGCCAGTTTGGGCCGCTCAAAAGTCCAGTCGCCCACGGTAATCGTGTCGGCCATATGGCC  240
               ||||||||||||||||||||||||||||||||||||||||||||||||||||||||||||
Sbjct  370755  CGCCAGTTTGGGCCGCTCAAAAGTCCAGTCGCCCACGGTAATCGTGTCGGCCATATGGCC  370696

Query  241     CCACATTTTGGCTTTTGCCGTATCCGGGCTGGTCGCGACGGTAAACTTCACGCTGCGGGA  300
               ||||||||||||||||||||||||||||||||||||||||||||||||||||||||||||
Sbjct  370695  CCACATTTTGGCTTTTGCCGTATCCGGGCTGGTCGCGACGGTAAACTTCACGCTGCGGGA  370636

Query  301     AACCGGCGTATTGATGTATGAATTGACGGGCACGATGCTTAACGGCGTCAGCAGCCCCAC  360
               ||||||||||||||||||||||||||||||||||||||||||||||||||||||||||||
Sbjct  370635  AACCGGCGTATTGATGTATGAATTGACGGGCACGATGCTTAACGGCGTCAGCAGCCCCAC  370576

Query  361     GCCCTTTTTCTGGGTGAGGATAATGGTCGCATCGCCGTTAGCGTCGGTGATCCCGTGATA  420
               ||||||||||||||||||||||||||||||||||||||||||||||||||||||||||||
Sbjct  370575  GCCCTTTTTCTGGGTGAGGATAATGGTCGCATCGCCGTTAGCGTCGGTGATCCCGTGATA  370516

Query  421     AACCTGCCCGGCAACGTAGCCCACGTCGTCAAACTGCATCTCGCCGTTGCTGGTATCGGT  480
               ||||||||||||||||||||||||||||||||||||||||||||||||||||||||||||
Sbjct  370515  AACCTGCCCGGCAACGTAGCCCACGTCGTCAAACTGCATCTCGCCGTTGCTGGTATCGGT  370456

Query  481     AAAGCCGGTCGTCAGCCCGTCACGCCGCCTGCCGTTCGCCATGGTGATGGTGAAGTCGGT  540
               ||||||||||||||||||||||||||||||||||||||||||||||||||||||||||||
Sbjct  370455  AAAGCCGGTCGTCAGCCCGTCACGCCGCCTGCCGTTCGCCATGGTGATGGTGAAGTCGGT  370396

Query  541     ATTGCCGATCGCTATGCCGTTAAGGGCGTTGCGCGAATGGATGTTCATCTTAACCTGTTC  600
               ||||||||||||||||||||||||||||||||||||||||||||||||||||||||||||
```

```
              ||||||||||||||||||||||||||||||||||||||||||||||||||||||||||||
Sbjct  370395  ATTGCCGATCGCTATGCCGTTAAGGGCGTTGCGCGAATGGATGTTCATCTTAACCTGTTC  370336
Query  601     ACCCACTTTGGCGTCGGCGTTTTTGATCACCGGGTCGTCGTTGAAGTAGACGTCCAGCAC  660
               ||||||||||||||||||||||||||||||||||||||||||||||||||||||||||||
Sbjct  370335  ACCCACTTTGGCGTCGGCGTTTTTGATCACCGGGTCGTCGTTGAAGTAGACGTCCAGCAC  370276
Query  661     CGCCACCATTTCATTGGCCGAGCAGGTCAGGTAGTTGGGCGTATCGGTAAATTTGCCGCT  720
               ||||||||||||||||||||||||||||||||||||||||||||||||||||||||||||
Sbjct  370275  CGCCACCATTTCATTGGCCGAGCAGGTCAGGTAGTTGGGCGTATCGGTAAATTTGCCGCT  370216
Query  721     ATCGCCGTTTTCGAGGTTGACGTGTACCGTCCCGGAGGCGTCGCTGTCGGCGGCGATGAA  780
               ||||||||||||||||||||||||||||||||||||||||||||||||||||||||||||
Sbjct  370215  ATCGCCGTTTTCGAGGTTGACGTGTACCGTCCCGGAGGCGTCGCTGTCGGCGGCGATGAA  370156
Query  781     ATAATCTTCA  790
               ||||||||||
Sbjct  370155  ATAATCTTCA  370146
```

Salmonella enterica subsp. enterica serovar Typhimurium strain FORC50 chromosome, complete genome
Sequence ID: **CP019383.1** Length: 4673894 Number of Matches: 1
Range 1: 1273445 to 1274234

Score	Expect	Identities	Gaps	Strand	Frame
1459 bits(790)	0.0()	790/790(100%)	0/790(0%)	Plus/Minus	

Features:

```
Query  1        AATGCTGTGAATCTTGCCGCCGCTGTTGGCGTTATACAGCGCCTCAAGCTGGTCGATGCG  60
                ||||||||||||||||||||||||||||||||||||||||||||||||||||||||||||
Sbjct  1274234  AATGCTGTGAATCTTGCCGCCGCTGTTGGCGTTATACAGCGCCTCAAGCTGGTCGATGCG  1274175
Query  61       CGGCAGGCGGTTCGCCGCGCAGCCGCCCGCATCCGGGTTGCCCACCGCGTCGCTATGGGC  120
                ||||||||||||||||||||||||||||||||||||||||||||||||||||||||||||
Sbjct  1274174  CGGCAGGCGGTTCGCCGCGCAGCCGCCCGCATCCGGGTTGCCCACCGCGTCGCTATGGGC  1274115
Query  121      CACGCGCGCCCAGGTCTCATTGCTCTCGTCCTGGGTACGCAGCGGGTTGCTCACTTCACC  180
                ||||||||||||||||||||||||||||||||||||||||||||||||||||||||||||
Sbjct  1274114  CACGCGCGCCCAGGTCTCATTGCTCTCGTCCTGGGTACGCAGCGGGTTGCTCACTTCACC  1274055
Query  181      CGCCAGTTTGGGCCGCTCAAAAGTCCAGTCGCCCACGGTAATCGTGTCGGCCATATGGCC  240
                ||||||||||||||||||||||||||||||||||||||||||||||||||||||||||||
Sbjct  1274054  CGCCAGTTTGGGCCGCTCAAAAGTCCAGTCGCCCACGGTAATCGTGTCGGCCATATGGCC  1273995
Query  241      CCACATTTTGGCTTTTGCCGTATCCGGGCTGGTCGCGACGGTAAACTTCACGCTGCGGGA  300
                ||||||||||||||||||||||||||||||||||||||||||||||||||||||||||||
Sbjct  1273994  CCACATTTTGGCTTTTGCCGTATCCGGGCTGGTCGCGACGGTAAACTTCACGCTGCGGGA  1273935
Query  301      AACCGGCGTATTGATGTATGAATTGACGGGCACGATGCTTAACGGCGTCAGCAGCCCCAC  360
                ||||||||||||||||||||||||||||||||||||||||||||||||||||||||||||
Sbjct  1273934  AACCGGCGTATTGATGTATGAATTGACGGGCACGATGCTTAACGGCGTCAGCAGCCCCAC  1273875
Query  361      GCCCTTTTTCTGGGTGAGGATAATGGTCGCATCGCCGTTAGCGTCGGTGATCCCGTGATA  420
                ||||||||||||||||||||||||||||||||||||||||||||||||||||||||||||
Sbjct  1273874  GCCCTTTTTCTGGGTGAGGATAATGGTCGCATCGCCGTTAGCGTCGGTGATCCCGTGATA  1273815
Query  421      AACCTGCCCGGCAACGTAGCCCACGTCGTCAAACTGCATCTCGCCGTTGCTGGTATCGGT  480
                ||||||||||||||||||||||||||||||||||||||||||||||||||||||||||||
Sbjct  1273814  AACCTGCCCGGCAACGTAGCCCACGTCGTCAAACTGCATCTCGCCGTTGCTGGTATCGGT  1273755
Query  481      AAAGCCGGTCGTCAGCCCGTCACGCCGCCTGCCGTTCGCCATGGTGATGGTGAAGTCGGT  540
                ||||||||||||||||||||||||||||||||||||||||||||||||||||||||||||
Sbjct  1273754  AAAGCCGGTCGTCAGCCCGTCACGCCGCCTGCCGTTCGCCATGGTGATGGTGAAGTCGGT  1273695
Query  541      ATTGCCGATCGCTATGCCGTTAAGGGCGTTGCGCGAATGGATGTTCATCTTAACCTGTTC  600
                ||||||||||||||||||||||||||||||||||||||||||||||||||||||||||||
Sbjct  1273694  ATTGCCGATCGCTATGCCGTTAAGGGCGTTGCGCGAATGGATGTTCATCTTAACCTGTTC  1273635
Query  601      ACCCACTTTGGCGTCGGCGTTTTTGATCACCGGGTCGTCGTTGAAGTAGACGTCCAGCAC  660
                ||||||||||||||||||||||||||||||||||||||||||||||||||||||||||||
Sbjct  1273634  ACCCACTTTGGCGTCGGCGTTTTTGATCACCGGGTCGTCGTTGAAGTAGACGTCCAGCAC  1273575
Query  661      CGCCACCATTTCATTGGCCGAGCAGGTCAGGTAGTTGGGCGTATCGGTAAATTTGCCGCT  720
                ||||||||||||||||||||||||||||||||||||||||||||||||||||||||||||
Sbjct  1273574  CGCCACCATTTCATTGGCCGAGCAGGTCAGGTAGTTGGGCGTATCGGTAAATTTGCCGCT  1273515
Query  721      ATCGCCGTTTTCGAGGTTGACGTGTACCGTCCCGGAGGCGTCGCTGTCGGCGGCGATGAA  780
                ||||||||||||||||||||||||||||||||||||||||||||||||||||||||||||
Sbjct  1273514  ATCGCCGTTTTCGAGGTTGACGTGTACCGTCCCGGAGGCGTCGCTGTCGGCGGCGATGAA  1273455
Query  781      ATAATCTTCA  790
                ||||||||||
Sbjct  1273454  ATAATCTTCA  1273445
```

Salmonella enterica subsp. enterica serovar Enteritidis strain SJTUF10984 chromosome, complete genome
Sequence ID: **CP015526.1** Length: 4679791 Number of Matches: 1
Range 1: 2578966 to 2579755

Score	Expect	Identities	Gaps	Strand	Frame
1459 bits(790)	0.0()	790/790(100%)	0/790(0%)	Plus/Plus	

Features:

```
Query  1        AATGCTGTGAATCTTGCCGCCGCTGTTGGCGTTATACAGCGCCTCAAGCTGGTCGATGCG  60
                ||||||||||||||||||||||||||||||||||||||||||||||||||||||||||||
Sbjct  2578966  AATGCTGTGAATCTTGCCGCCGCTGTTGGCGTTATACAGCGCCTCAAGCTGGTCGATGCG  2579025
```

```
Query  61       CGGCAGGCGGTTCGCCGCGCAGCCGCCCGCATCCGGGTTGCCCACCGCGTCGCTATGGGC  120
                ||||||||||||||||||||||||||||||||||||||||||||||||||||||||||||
Sbjct  2579026  CGGCAGGCGGTTCGCCGCGCAGCCGCCCGCATCCGGGTTGCCCACCGCGTCGCTATGGGC  2579085

Query  121      CACGCGCGCCCAGGTCTCATTGCTCTCGTCCTGGGTACGCAGCGGGTTGCTCACTTCACC  180
                ||||||||||||||||||||||||||||||||||||||||||||||||||||||||||||
Sbjct  2579086  CACGCGCGCCCAGGTCTCATTGCTCTCGTCCTGGGTACGCAGCGGGTTGCTCACTTCACC  2579145

Query  181      CGCCAGTTTGGGCCGCTCAAAAGTCCAGTCGCCCACGGTAATCGTGTCGGCCATATGGCC  240
                ||||||||||||||||||||||||||||||||||||||||||||||||||||||||||||
Sbjct  2579146  CGCCAGTTTGGGCCGCTCAAAAGTCCAGTCGCCCACGGTAATCGTGTCGGCCATATGGCC  2579205

Query  241      CCACATTTTGGCTTTTGCCGTATCCGGGCTGGTCGCGACGGTAAACTTCACGCTGCGGGA  300
                ||||||||||||||||||||||||||||||||||||||||||||||||||||||||||||
Sbjct  2579206  CCACATTTTGGCTTTTGCCGTATCCGGGCTGGTCGCGACGGTAAACTTCACGCTGCGGGA  2579265

Query  301      AACCGGCGTATTGATGTATGAATTGACGGGCACGATGCTTAACGGCGTCAGCAGCCCCAC  360
                ||||||||||||||||||||||||||||||||||||||||||||||||||||||||||||
Sbjct  2579266  AACCGGCGTATTGATGTATGAATTGACGGGCACGATGCTTAACGGCGTCAGCAGCCCCAC  2579325

Query  361      GCCCTTTTTCTGGGTGAGGATAATGGTCGCATCGCCGTTAGCGTCGGTGATCCCGTGATA  420
                ||||||||||||||||||||||||||||||||||||||||||||||||||||||||||||
Sbjct  2579326  GCCCTTTTTCTGGGTGAGGATAATGGTCGCATCGCCGTTAGCGTCGGTGATCCCGTGATA  2579385

Query  421      AACCTGCCCGGCAACGTAGCCCACGTCGTCAAACTGCATCTCGCCGTTGCTGGTATCGGT  480
                ||||||||||||||||||||||||||||||||||||||||||||||||||||||||||||
Sbjct  2579386  AACCTGCCCGGCAACGTAGCCCACGTCGTCAAACTGCATCTCGCCGTTGCTGGTATCGGT  2579445

Query  481      AAAGCCGGTCGTCAGCCCGTCACGCCGCCTGCCGTTCGCCATGGTGATGGTGAAGTCGGT  540
                ||||||||||||||||||||||||||||||||||||||||||||||||||||||||||||
Sbjct  2579446  AAAGCCGGTCGTCAGCCCGTCACGCCGCCTGCCGTTCGCCATGGTGATGGTGAAGTCGGT  2579505

Query  541      ATTGCCGATCGCTATGCCGTTAAGGGCGTTGCGCGAATGGATGTTCATCTTAACCTGTTC  600
                ||||||||||||||||||||||||||||||||||||||||||||||||||||||||||||
Sbjct  2579506  ATTGCCGATCGCTATGCCGTTAAGGGCGTTGCGCGAATGGATGTTCATCTTAACCTGTTC  2579565

Query  601      ACCCACTTTGGCGTCGGCGTTTTTGATCACCGGGTCGTCGTTGAAGTAGACGTCCAGCAC  660
                ||||||||||||||||||||||||||||||||||||||||||||||||||||||||||||
Sbjct  2579566  ACCCACTTTGGCGTCGGCGTTTTTGATCACCGGGTCGTCGTTGAAGTAGACGTCCAGCAC  2579625

Query  661      CGCCACCATTTCATTGGCCGAGCAGGTCAGGTAGTTGGGCGTATCGGTAAATTTGCCGCT  720
                ||||||||||||||||||||||||||||||||||||||||||||||||||||||||||||
Sbjct  2579626  CGCCACCATTTCATTGGCCGAGCAGGTCAGGTAGTTGGGCGTATCGGTAAATTTGCCGCT  2579685

Query  721      ATCGCCGTTTTCGAGGTTGACGTGTACCGTCCCGGAGGCGTCGCTGTCGGCGGCGATGAA  780
                ||||||||||||||||||||||||||||||||||||||||||||||||||||||||||||
Sbjct  2579686  ATCGCCGTTTTCGAGGTTGACGTGTACCGTCCCGGAGGCGTCGCTGTCGGCGGCGATGAA  2579745

Query  781      ATAATCTTCA  790
                ||||||||||
Sbjct  2579746  ATAATCTTCA  2579755
```

Salmonella enterica subsp. enterica serovar Enteritidis strain SJTUF10978 chromosome, complete genome
Sequence ID: **CP015524.1** Length: 4679990 Number of Matches: 1
Range 1: 2579178 to 2579967

Score	Expect	Identities	Gaps	Strand	Frame
1459 bits(790)	0.0()	790/790(100%)	0/790(0%)	Plus/Plus	

Features:

```
Query  1        AATGCTGTGAATCTTGCCGCCGCTGTTGGCGTTATACAGCGCCTCAAGCTGGTCGATGCG  60
                ||||||||||||||||||||||||||||||||||||||||||||||||||||||||||||
Sbjct  2579178  AATGCTGTGAATCTTGCCGCCGCTGTTGGCGTTATACAGCGCCTCAAGCTGGTCGATGCG  2579237

Query  61       CGGCAGGCGGTTCGCCGCGCAGCCGCCCGCATCCGGGTTGCCCACCGCGTCGCTATGGGC  120
                ||||||||||||||||||||||||||||||||||||||||||||||||||||||||||||
Sbjct  2579238  CGGCAGGCGGTTCGCCGCGCAGCCGCCCGCATCCGGGTTGCCCACCGCGTCGCTATGGGC  2579297

Query  121      CACGCGCGCCCAGGTCTCATTGCTCTCGTCCTGGGTACGCAGCGGGTTGCTCACTTCACC  180
                ||||||||||||||||||||||||||||||||||||||||||||||||||||||||||||
Sbjct  2579298  CACGCGCGCCCAGGTCTCATTGCTCTCGTCCTGGGTACGCAGCGGGTTGCTCACTTCACC  2579357

Query  181      CGCCAGTTTGGGCCGCTCAAAAGTCCAGTCGCCCACGGTAATCGTGTCGGCCATATGGCC  240
                ||||||||||||||||||||||||||||||||||||||||||||||||||||||||||||
Sbjct  2579358  CGCCAGTTTGGGCCGCTCAAAAGTCCAGTCGCCCACGGTAATCGTGTCGGCCATATGGCC  2579417

Query  241      CCACATTTTGGCTTTTGCCGTATCCGGGCTGGTCGCGACGGTAAACTTCACGCTGCGGGA  300
                ||||||||||||||||||||||||||||||||||||||||||||||||||||||||||||
Sbjct  2579418  CCACATTTTGGCTTTTGCCGTATCCGGGCTGGTCGCGACGGTAAACTTCACGCTGCGGGA  2579477

Query  301      AACCGGCGTATTGATGTATGAATTGACGGGCACGATGCTTAACGGCGTCAGCAGCCCCAC  360
                ||||||||||||||||||||||||||||||||||||||||||||||||||||||||||||
Sbjct  2579478  AACCGGCGTATTGATGTATGAATTGACGGGCACGATGCTTAACGGCGTCAGCAGCCCCAC  2579537

Query  361      GCCCTTTTTCTGGGTGAGGATAATGGTCGCATCGCCGTTAGCGTCGGTGATCCCGTGATA  420
                ||||||||||||||||||||||||||||||||||||||||||||||||||||||||||||
Sbjct  2579538  GCCCTTTTTCTGGGTGAGGATAATGGTCGCATCGCCGTTAGCGTCGGTGATCCCGTGATA  2579597

Query  421      AACCTGCCCGGCAACGTAGCCCACGTCGTCAAACTGCATCTCGCCGTTGCTGGTATCGGT  480
                ||||||||||||||||||||||||||||||||||||||||||||||||||||||||||||
Sbjct  2579598  AACCTGCCCGGCAACGTAGCCCACGTCGTCAAACTGCATCTCGCCGTTGCTGGTATCGGT  2579657

Query  481      AAAGCCGGTCGTCAGCCCGTCACGCCGCCTGCCGTTCGCCATGGTGATGGTGAAGTCGGT  540
                ||||||||||||||||||||||||||||||||||||||||||||||||||||||||||||
Sbjct  2579658  AAAGCCGGTCGTCAGCCCGTCACGCCGCCTGCCGTTCGCCATGGTGATGGTGAAGTCGGT  2579717

Query  541      ATTGCCGATCGCTATGCCGTTAAGGGCGTTGCGCGAATGGATGTTCATCTTAACCTGTTC  600
                ||||||||||||||||||||||||||||||||||||||||||||||||||||||||||||
Sbjct  2579718  ATTGCCGATCGCTATGCCGTTAAGGGCGTTGCGCGAATGGATGTTCATCTTAACCTGTTC  2579777

Query  601      ACCCACTTTGGCGTCGGCGTTTTTGATCACCGGGTCGTCGTTGAAGTAGACGTCCAGCAC  660
                ||||||||||||||||||||||||||||||||||||||||||||||||||||||||||||
Sbjct  2579778  ACCCACTTTGGCGTCGGCGTTTTTGATCACCGGGTCGTCGTTGAAGTAGACGTCCAGCAC  2579837
```

Supplementary FIGURE S2 | BLAST search results using *Salmonella* Gallinarum *ratA ROD* nucleotide sequence (GenBank accession no. AM933173.1 segment 2636413-2636983) against the nucleotide collection (nr/nt) database. The maximum number of aligned sequences to display was set to the maximum value of 20,000, and the other parameters were set to default values. The results showed that *ratA ROD* fragment exists only in *S.* Gallinarum and other *Salmonella* serovars, which could be exploited to distinguish *S.* Pullorum from them.
